# High-fidelity imaging of amyloid-beta deposits with an ultrasensitive fluorescent probe facilitates the early diagnosis and treatment of Alzheimer's Disease

**DOI:** 10.7150/thno.68743

**Published:** 2022-02-28

**Authors:** Rongrong Tao, Ning Wang, Tianruo Shen, Yuhang Tan, Yong Ren, Wenjing Wei, Meihua Liao, Davin Tan, Chunzhi Tang, Nenggui Xu, Huan Wang, Xiaogang Liu, Xin Li

**Affiliations:** 1 Medical College of Acupuncture-Moxibustion and Rehabilitation, Guangzhou University of Chinese Medicine, Guangzhou, Guangdong 510006, China; 2 College of Pharmaceutical Sciences, Zhejiang University, Hangzhou, Zhejiang 301158, China; 3 Fluorescence Research Group, Singapore University of Technology and Design, 8 Somapah Road, Singapore 487372, Singapore; 4 College of Life Science and Technology, Dalian University, Dalian, Liaoning 116622, China

**Keywords:** fluorescent probe, fluorescent imaging, amyloid-beta, Alzheimer's disease, intramolecular charge transfer

## Abstract

**Background:** Imaging amyloid-beta (Aβ) deposits with high fidelity in naturally aging brains is crucial for the early diagnosis of Alzheimer's disease (AD). However, this is impeded by the lack of highly sensitive probes.

**Methods:** By conducting computational modelling to quantitatively fine-tune the twisted intramolecular charge transfer (TICT) tendency of Thioflavin T (**ThT**) analogues, we developed an ultrasensitive probe **AH-2**. **AH-2** retained the binding affinity and binding mode of **ThT** towards Aβ deposits, and exhibited *ca* 10-fold less background fluorescence and 5-10 folds of improved signal-to-background contrast upon binding Aβ deposits. These desirable features endowed **AH-2** the sensitivity to detect Aβ deposition in naturally aging wild-type mice.

**Results: AH-2** imaging revealed that Aβ puncta signals appeared near the nuclei in young mice and spread through the intracellular and extracellular compartments in older mice. Moreover, Aβ deposits were observed to emerge earlier in mice cerebral cortex than in the hippocampus region. Given this desirable sensitivity and good spatiotemporal resolution, **AH-2** was successfully applied in the preclinical evaluation of Aβ-targeted treatment by melatonin.

**Conclusions:** We expect that **AH-2** is promising for early diagnosis of AD and will serve as a sensitive tool for studying Aβ-related AD pathology.

## Introduction

Alzheimer's disease (AD) is the leading cause of neurological disorders in the aging population. It is characterized by continuous memory loss and progressive decline of cognitive abilities, eventually resulting in the death of patients 1, 2. In the United States alone, AD caused 122,019 deaths in 2018, and cost $244 billion in patient care in 2019, in addition to unmeasurable physical and mental distress to both patients and caregivers 3. Unfortunately, no effective treatment is currently available to restore AD-related neurological deficits in clinical cases, which is largely attributed to the delayed intervention and the complexity of AD pathogenesis 4, 5. Early detection and accurate diagnosis are thus crucial to the success of pre-symptomatic intervention initiation and the improvement of treatment outcomes 6.

To obtain accurate information with predictive or early diagnostic value, researchers have been targeting the cerebral deposition of amyloid-beta (Aβ) peptides, a pathological hallmark of AD that can occur decades before the onset of dementia 7, 8. Previous studies have revealed that Aβ deposition, hippocampal atrophy, and episodic memory impairment occurred sequentially in elderly subjects, with Aβ deposition being the initial event in the cascade 9, 10. These results suggest that detecting cerebral Aβ deposition with high fidelity would offer credible information to support the early diagnosis of AD. However, an extensive breakthrough in the field is still needed for the effective imaging of Aβ deposition before the onset or in the pre-symptomatic stage of AD.

Currently, there are two molecular imaging modalities for the non-invasive detection of Aβ deposits, namely radionuclide imaging and fluorescence imaging 11. Radionuclide imaging includes single-photon emission computed tomography (SPECT) and positron emission computed tomography (PET) 12. The radioactive nature of SPECT and PET, coupled with their heavy reliance on radiochemistry facilities, makes these techniques less ideal and limits their widespread applicability. In contrast, fluorescence imaging represents a much safer and more affordable alternative to realize the *in vivo* imaging of Aβ plaques. In this regard, various fluorescent probes selectively staining Aβ deposits have been developed 13-17, with Congo Red and Thioflavin T (**ThT**) being the key representatives. Whilst some reported probes have realized the *in vivo* imaging of Aβ deposits in AD-modelled transgenic mice 18-23, highly sensitive probes remain underexplored that are capable of imaging Aβ deposits in naturally aged wide-type mice which express only a trace amount of Aβ deposits and represent the pre-symptomatic stage of AD. The ability to effectively image Aβ deposition in the normal brain aging process is essential to understand Aβ-related dementia pathogenesis and could serve as a gold standard for predicting the onset of AD 24, 25. However, accurately mapping Aβ deposits in normal mice before the onset of AD demands a probe with ultra-sensitivity and high fidelity.

Herein, by computationally modelling to fine-tune the twisted intramolecular charge transfer (TICT) tendency of **ThT** 26, we developed an ultrasensitive probe **AH-2** for detecting Aβ deposits. Unbound **AH-2** emitted negligible fluorescence in an aqueous solution, *ca.* 10-fold less than the parent **ThT** structure. However, **AH-2** displayed dramatically enhanced fluorescence in the range of 550-650 nm upon binding to Aβ deposits, and this fluorogenic response was relatively specific towards Aβ deposits. Accordingly, **AH-2** afforded desirable signal-to-noise ratio and enabled three-dimensional (3D) imaging of Aβ deposits with good spatial resolution in the aging brains of mice both *in vitro* and *in vivo*. Our experiments revealed that the number of Aβ deposits increased in an age-dependent manner in the wide-type mice, and significant Aβ deposits were observed in wide-type mice as early as 8 months old. The spatial distribution pattern of Aβ deposits in mice aging process was also recorded facilitated by **AH-2** imaging. Moreover, **AH-2** was successfully applied to assess the efficacy of anti-AD treatments and revealed the great potency of melatonin to decrease cerebral Aβ plaques. These results highlighted the success of this computer-aided probe design strategy and demonstrated **AH-2** as a promising tool for early AD diagnosis and treatment evaluation.

## Materials and Methods

### Materials

Unless otherwise stated, all reagents and chemicals for imaging experiments were purchased from Sigma Aldrich. Human Aβ_1-42_ monomer was obtained from Chinese Peptide Company (Lot: AMYD-003). Thioflavin T (ThT) dye (HY-D0218) was purchased from MedChemExpress (Shanghai, China). Primary antibodies PKC (2056S), p-PKC (9375S), and HRP-Linked secondary antibody (7074S, 7076S) for Western-blot analysis were purchased from Cell Signal Technology (Massachusetts, US). GAPDH (70-Mab5465-040) was bought from Multi-Sciences Biotech (Hangzhou, China). Primary antibody to Aβ_1-42/1-40_ (NBP2-13075) was bought from Novus Biologicals (Minnesota, US). The Alexa-fluor 647 mouse IgG (A-31571) was purchased from Thermo Fisher Scientific (Massachusetts, US). Antifade mounting medium containing DAPI (H1200) was obtained from Vector Labs (California, US).

### Probe synthesis and structure elucidation

Methods for probe synthesis and for structure elucidation were described in the supporting information.

### Animals and drug treatment

All animal experiments were conducted following the experimental animal care and use guidelines of the National Institutes of Health (NIH, USA). Experiment protocols and procedures were approved by the Committee for Animal Experiments at Guangzhou University of Chinese Medicine in China (permit code: SYXK (YUE) 2017-0179). AD-modelled 3xTg AD mice and 5xFAD mice were kindly provided by Song lab in Guangzhou University of Chinese Medicine. Male and female mice were mated and bred in stable conditions in terms of temperature, humidity, and 12 h light/dark cycle. Standard laboratory diet and water were provided for the mice. The male age-matched wild type, 3xTg AD and 5xFAD mice at specific ages ranging from 2 months to 13 months were used in the present study for* in vivo* two-photon microscope imaging, immunofluorescence staining, and Western blot analysis. For melatonin treatment, mice at age of 12 months were administrated with melatonin (M5250, Sigma Aldrich) at a daily dose of 10 mg/kg for 4 weeks. Melatonin was dissolved in ethanol and then diluted by water to 1% ethanol.

### Immunofluorescence and Western blot analysis

Detailed methods were described in the supporting information.

### *In vivo* two-photon imaging

To image Aβ aggregates *in vivo*, two-photon microscope imaging was performed using an upright two-photon laser scanning microscope Nikon A1R MP equipped with Sapphire laser (Coherent, chameleon vision II) and Nikon 25 × 1.1 NA water-immersion microscope objective lens. The 3xTg AD mice (2, 8, 13 months old, male) and age-matched WT mice (2, 8, 13 months old, male) were anesthetized with 2.5 % isoflurane in 1.5 L/min oxygen flow, and preparation for cranial imaging window surgery was previously described 27. The anesthetized mice were imaged through a craniotomy window with a 4 mm diameter, centered at stereotaxic coordinates 2.5 mm caudal to bregma and 2.5 mm lateral to the midline. After **AH-2** administration, the glass cover lid (6 mm diameter) with a metal frame (10 mm diameter) was glued to the skull to cover the craniotomy window before imaging. To monitoring Aβ aggregation in the brain of 3xTg AD model mice and age-matched WT mice, the **AH-2** signal was collected from emission at 580 nm upon the excitation at 860 nm. The images were taken at depth of 100-150 μm below the cortical surface. The images were captured at a solution of 512 × 512 pixels resolution. Image J software was used for image analysis.

### Statistics

The data were present graphically and analysed statistically with one-way analysis of variance (ANOVA) followed by a post hoc Tukey's test or Dunnett's comparison to control, or two-way ANOVA analysis for groups during aging. All data were expressed as mean ± SEM, and statistical significance was marked when *p* < 0.05.

## Results and Discussion

### Computer-aided probe design

**Figure 1:**
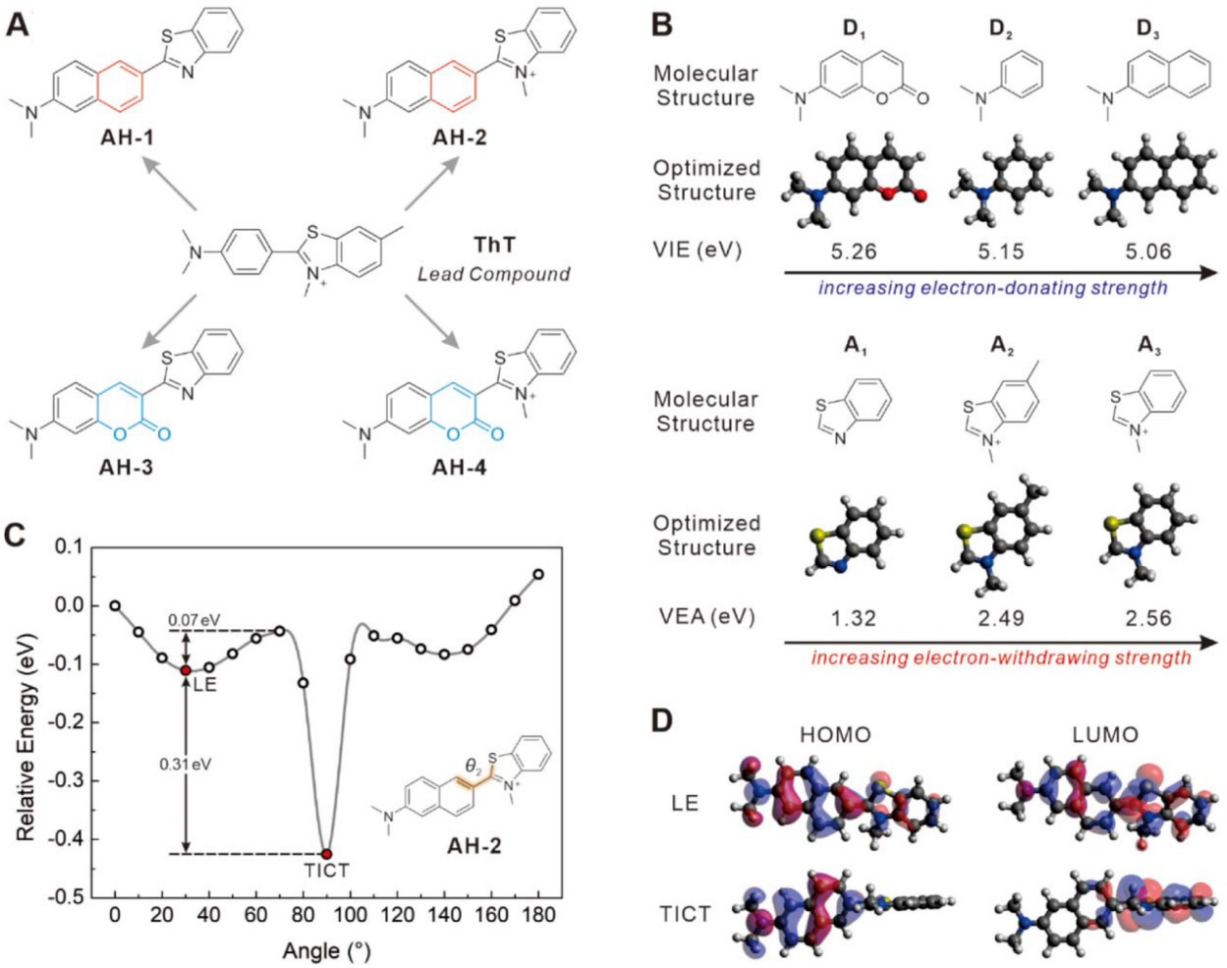
** Molecular design strategy. (A)** Molecular structures of ThT, AH-1, AH-2, AH-3, and AH-4. **(B)** Calculated vertical ionization energies (VIE, eV) of donors D1-D3 and calculated vertical electron affinities (VEA, eV) of acceptors A1-A3. **(C)** Calculated potential energy surface of AH-2 as a function of *θ_2_* in water. (D) HOMOs and LUMOs of AH-2 in the LE and TICT states in water.

Currently, **ThT** is probably the most popular fluorescent dye used for staining Aβ fibrils (Figure 1A). **ThT** is weakly fluorescent in aqueous solutions but exhibits an enhanced emission at 482 nm upon binding to Aβ plaques. This fluorogenic property makes it particularly attractive for the no-wash labelling of Aβ plaques. It is generally accepted that the fluorogenicity of **ThT** resulted from the modulation of TICT. The free rotation of **ThT** in a non-viscous solution enables the formation of the TICT state upon photoexcitation, which quenches its fluorescence 28. Upon binding with amyloid fibrils at the side chain channels along the long axis of amyloid fibrils 29, 30, the TICT rotation of **ThT** is inhibited, leading to strong emission 31. **ThT** is therefore a good starting point to develop ultrasensitive probes for staining Aβ fibrils in naturally-aged wide-type mice. To further improve the signal-to-background imaging contrast of **ThT**, it is essential to minimize background fluorescence that originates from the unbound probe 32. Hence, we set out to tune the TICT effect of **ThT** by adjusting the electron-donating group (EDG) and the electron-withdrawing group (EWG), respectively. In this context, the aniline part of **ThT** was replaced by a naphthalene amine or a coumarine amine moiety to tune its electron-donating ability, and four derivatives (**AH-1**,** AH-2**,** AH-3**, and** AH-4**) were designed (Figure 1A).

To assess the TICT tendency of these probes, we first analysed the electron-donating/withdrawing strength of their main building blocks, including donors **D1**-**D3** and acceptors **A1**-**A3**. This is because increasing the donor electron-donating strength and/or the acceptor electron-withdrawing strength tends to enhance the TICT tendency, thus decreasing background emissions and improving the overall signal-to-noise ratios. According to our calculated vertical ionization energy in vacuo (Figure 1B and S1), the electron-donating strength increases in the order of **D1** < **D2** < **D3**. Among the three acceptors, calculated vertical electron affinity revealed that the electron-withdrawing strength follows the order of **A1** < **A2** < **A3** (Figure 1B). These results suggest that **AH-2** (or **D3**+**A3**) possesses the strongest push-pull effect among all D-A combinations, thus affording a higher TICT tendency than other compounds, such as **ThT** (or **D2**+**A2**), **AH-1** (or **D3**+**A1**), **AH-3** (or **D1**+**A1**), and **AH-4** (**D1**+**A3**).

Indeed, modelling of the excited state potential energy surface (PES) revealed that among **AH-1**, **AH-2**, **AH-3,** and **AH-4**, which possess similar molecular sizes, only **AH-2** displayed a stable TICT state, with a small energy barrier of 0.07 eV and a strong driving force of 0.31 eV (Figure 1C). Notably, while the quasi-planar locally excited (LE) state of **AH-2** exhibits a large oscillator strength (*f* = 1.5537), the twisted conformation of **AH-2** (*θ* = ~90°) possesses a nearly zero oscillator strength (*f* = 0.0058), with complete charge separation between the donor and acceptor fragments (Figure 1D). These features are consistent with the TICT mechanism and suggest negligible background emissions from **AH-2** in a non-viscous environment, due to considerable TICT formation rates.

In stark contrast, PES calculations show that only a stable LE state exists in **AH-1**, **AH-3**, and **AH-4**, and the twisted conformations of these compounds are not stable (Figure S1). In other words, these three compounds are not prone to TICT formations and would exhibit strong background emissions. Moreover, even though previous studies have shown that **ThT** could form the TICT state upon photoexcitation 33, based on our calculations,** ThT** possesses a weaker push-pull strength than our designed **AH-2** probe.

These modelling experiments predict that amongst all the probe candidates, AH-2 would exhibit the strongest TICT tendency, the lowest background emission, and the highest fluorescence turn-on ratio upon binding with Aβ fibrils.

### Confirming the improved sensitivity of AH-2 to Aβ deposits by solution-based assay

**Figure 2:**
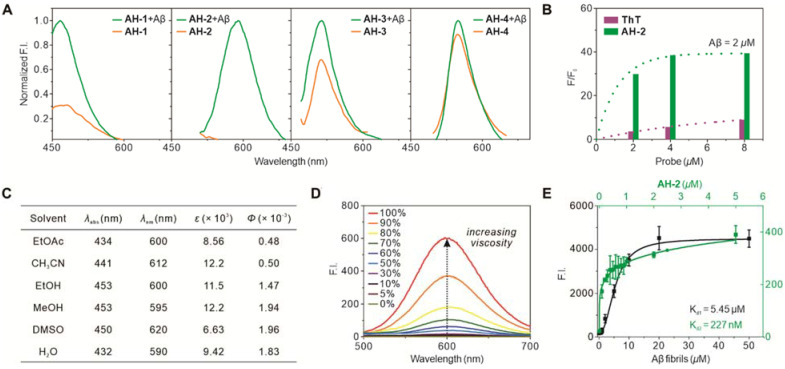
** Photophysical characterization of Aβ fibrils-specific response of AH-2. (A)** Fluorescence-response of probes **AH-1**—**AH-4** to Aβ fibrils. The concentrations of probes were kept at 5 µM, while that of Aβ fibrils was kept at 2.5 µM. The fluorescence spectra of each probe before binding Aβ fibrils were normalized to its emission after the treatment of Aβ fibrils. **(B)** Fluorescence increment of probe **AH-2** (at 587 nm) in comparison to **ThT** (at 480 nm) in response to Aβ fibrils. Each probe was kept at 2, 4, or 8 µM; Aβ fibrils were kept at 2 µM. *F* was the intensity of the probe treated with Aβ fibrils; while *F*_0_ was the probe intensity at the indicated concentration without Aβ fibrils treatment. **(C)** Photophysical property of **AH-2** in solvents of various polarities. **(D)** Fluorescence spectra of **AH-2** in PBS with various proportional glycerol to adjusting solution viscosity.** (E)** Binding affinity plot between **AH-2** and Aβ fibrils. *K_d1_* plot was carried out by setting **AH-2** at a fixed concentration of 100 nM and varying concentrations of Aβ (1-42) aggregates. *K_d2_* plot was carried out by setting Aβ (1-42) aggregates at a fixed concentration of 100 nM and varying concentrations of **AH-2**. Data in A, B, and D were recorded on a fluorescent spectrometry while those in E were recorded on a microplate reader.

To test the reliability of the afore-mentioned probe design strategy, probes **AH-1, AH-2, AH-3,** and** AH-4** were subsequently synthesized (Schemes S1 and S2). Their photophysical responses towards Aβ deposits were tested accordingly. The Aβ deposits were prepared from human Aβ (1-42) peptides and confirmed by transmission electron microscopy, which showed fibril formation (Figure S2). Then the four probes were individually recorded for their fluorescence spectra before and after exposure to Aβ fibrils. As shown in the normalized emission spectra (Figure 2A), only probe **AH-2** demonstrated a robust fluorescence enhancement upon the treatment of Aβ fibrils, whereas the increase for probe** AH-1, AH-3, and AH-4** was generally below 3-fold. This is in good agreement with the computational results which showed that these three compounds are not prone to TICT formations and thus displayed strong background emissions and low signal-to-noise ratios.

Next, we compared the sensitivity of **AH-2** with that of **ThT**. Various concentrations of **AH-2** and **ThT** were treated with Aβ fibrils (2 µM), and their fibril-triggered fluorescence response was recorded. **AH-2** displayed a much higher increase of fluorescence (F/F_0_) than **ThT** (Figure 2B), which denotes its improved signal-to-background contrast by *ca* 5-10 times. This is largely attributed to the decreased background emissions, underscoring the reliability of our computer-aided probe design strategy.

To confirm the TICT mechanism for the fluorogenic response of **AH-2** to Aβ fibrils, its emission spectra in solvents of various polarities or viscosities were recorded. As shown in Figure 2C and S3, **AH-2** emitted extremely weak in typical organic solvents, suggesting that surrounding polarity posed little effect on its fluorescence brightness. While in a PBS solution containing various proportions of glycerol, the fluorescence of **AH-2** intensified as the solvent viscosity increased by up to 750 times (Figure 2D and S4). This phenomenon suggested that inhibition of the free rotation of **AH-2** was the major contributor to the fluorogenic response, in accord with the TICT mechanism.

We also measured the binding constants (*K_d_*) between AH-2 and the Aβ fibrils, using two assay formats 34. First, the AH-2 concentration was fixed at 100 nM and treated with varying concentrations of Aβ fibrils. Plotting the fluorescence intensity against Aβ fibril concentrations gave a logistic regression fit, in which the *K_d1_* was determined to be 5.45 µM (Figure 2E). In the second format, Aβ fibril concentration was fixed at 100 nM and treated with varying concentrations of AH-2. Similarly, the resulting fluorescence intensity plot resulted in a logistic regression fit, and *K_d2_* was determined to be 227 nM (Figure 2E). These two *K_d_* values were similar to those of ThT 34, suggesting that the structure modification caused little effect on probe binding with Aβ fibrils. These two *K_d_* values can also be used to estimate the number of AH-2 binding sites on the Aβ fibrils 34. Since the Aβ deposit concentration herein is represented by the concentration of Aβ (1-42) peptide monomers before aggregation, according to literature 34, our obtained *K_d1_/K_d2_* ratio estimates one AH-2 binding site per 24 Aβ peptide monomers. This binding pattern is also similar to that of ThT, which suggests that probe AH-2 retains the same binding mode as ThT towards Aβ fibrils, but with improved sensitivity.

Aβ oligomers were reported to be the most toxic state among the various types of Aβ assemblies. To test whether probe AH-2 would be active towards Aβ oligomers, a solution of AH-2 was treated with Aβ oligomers and a dramatic fluorescence intensification was observed (Figure S5). Noteworthy, the emission spectra of AH-2 caused by Aβ oligomers differed from that caused by Aβ fibrils by a ca. 10-16 nm red-shift (Figure S6), which was presumably due to the different structures of Aβ oligomers and Aβ fibrils. We also confirmed that the fluorogenic response of AH-2 was specific to Aβ fibrils and is independent of other commonly-found biological analytes such as amino acids, metal cations (Figure S7). Though the benzothiazole moiety in AH-2 is structurally highly electron-deficient, it is relatively stable towards biological nucleophiles such as Cys and GSH (Figure S7). We also confirmed that the presence of Cys caused little interference on the sensitivity of AH-2 towards Aβ oligomers (Figure S8). Moreover, AH-2 also demonstrated the desirable photostability prerequisite for imaging experiments. When the fluorescence of AH-2 was triggered on by the treatment of Aβ fibrils, the continuous excitation caused little quenching effect on the intensity (Figure S9).

### Testing the selectivity and sensitivity of AH-2 in brain slices

After confirming the desirable performance of **AH-2** in the solution-based assay and before going forward for the imaging experiments, we tested the cytotoxicity of **AH-2**. It was shown that **AH-2** was quite safe under the working concentration of 5 µM (Figure S10). We then further tested its selectivity and sensitivity to endogenous Aβ deposits in AD mice brain slices. 5xFAD mice, 3xTg AD mice and APP/PS1 mice are wildly used in AD-related studies as well-recognized AD-modelled trans-genetic mice 35. However, the timeline accelerating AD onset and progression differs in these three strains of AD mice, with 5xFAD mice developing AD-like pathology within shortest latency while APP/PS1 mice developing AD-like pathology within longest latency. Herein, we used 5xFAD mice and 3xTg mice as the AD model, and wild-type mice were set as control for experiments.

**Figure 3:**
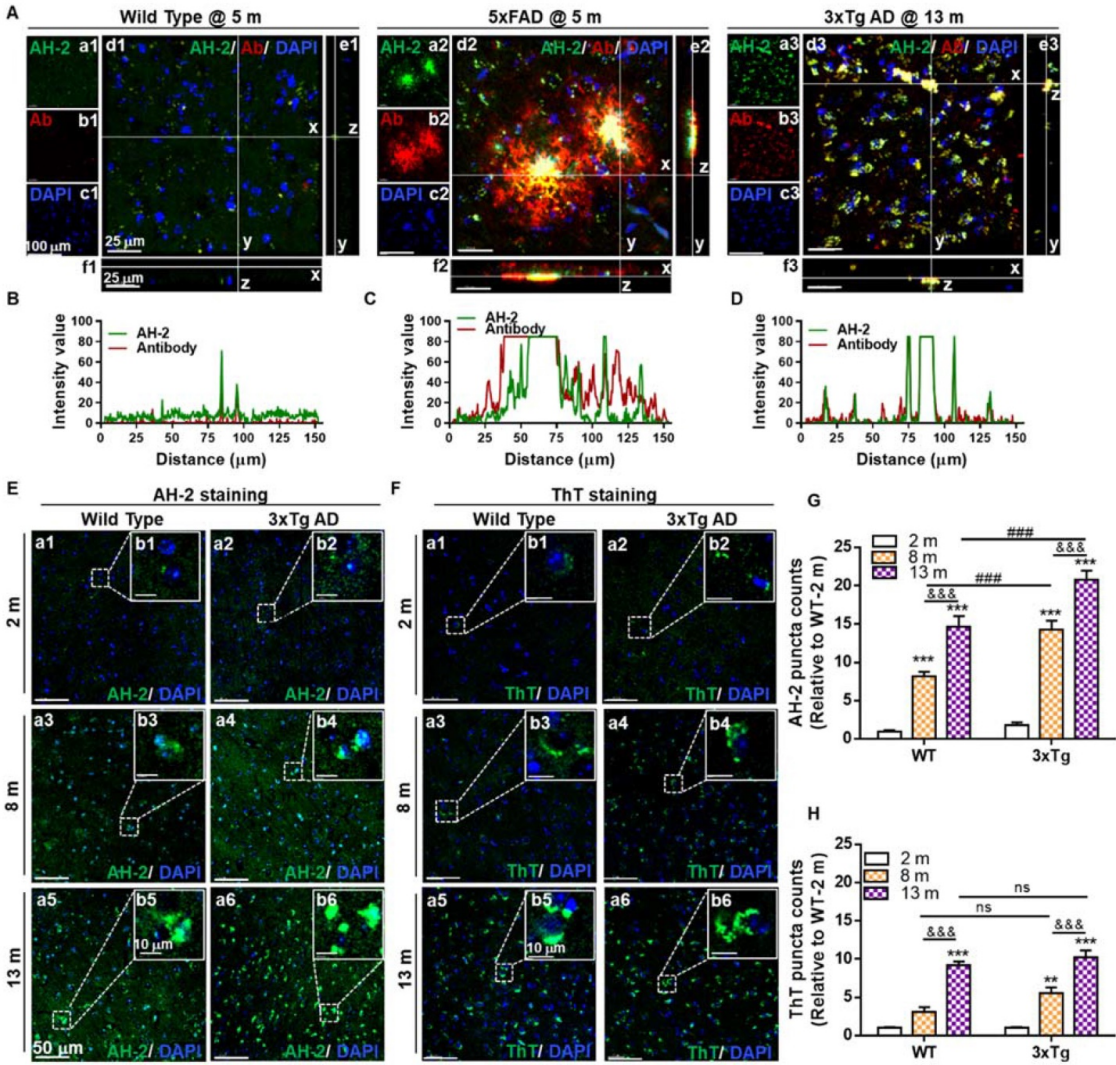
** Specificity and sensitivity test for probe AH-2 to Aβ deposits in mice brain slices. (A)** Representative confocal images of Aβ deposits labelled by** AH-2** and Aβ_1-42/1-40_ antibody in the brain cortex of wild-type mice, 5xFAD mice, and 3xTg AD mice. The orthogonal projections of Z-stack images (d1-d3) to y-z (e1-e3) and x-z (f1-f3) further demonstrated the co-localization of **AH-2** signal and Aβ antibody signal. **(B-D)** The fluorescence distributions of Aβ antibody signal (red line) and **AH-2** signal (green line) along line x in d1-d3. **(E, F)** Representative images of **AH-2** (E) and **ThT** (F) staining age-dependent Aβ accumulation in the cortex of wild-type mice and 3xTg mice at ages of 2, 8, and 13 months. Magnified images of boxed regions were displayed in images b1-b6 respectively. **(G, H)** Quantification of** AH-2** or **ThT**-labeled Aβ deposits in the cortex of wild-type and 3xTg AD mice. Data were expressed as Mean ± SEM normalized to WT at 2 months. **p < 0.01, ***p < 0.001 versus 2 months group. &&&* p* < 0.001 versus 8 months group. ###*p* < 0.001 versus age-matched WT group. ns, no significance. *N* = 5 mice. **AH-2**: green channel, λ_ex_ = 488 nm, λ_em_ = 595 nm; Aβ_1-42/1-40_ antibody: red channel, λ_ex_ = 640 nm, λ_em_ = 700 nm. Cell nuclei indicated by DAPI: blue channel, λ_ex_ = 405 nm, λ_em_ = 450 nm.

Firstly, probe selectivity was verified by double-staining brain tissues from the cortex region with **AH-2** and Aβ_1-42/1-40_ specific antibodies. We first confirmed that there was no fluorescence crosstalk or crossover between the **AH-2** and antibody signals (Figure S11). Then double-staining experiments were carried out. Strong fluorescence from both **AH-2** (the green channel) and Aβ_1-42/1-40_ antibody (the red channel) in the 5xFAD mice at 5 months old and 3xTg AD mice at 13 months old was observed by confocal imaging (Figure 3A), while the signals in wild-type mice at 5 months old were very weak (Figure 3A). This agrees with the fact that either 5xFAD or 3xTg mice overexpress amyloid precursor protein (APP) to form β-amyloid (Aβ) compared with non-transgenic mice. The overlay of the green and red signals in the 5xFAD group or 3xTg AD group highlights the good selectivity of **AH-2** towards Aβ deposits. Orthogonal projection of Z stack images further demonstrated the co-localization of **AH-2** signals with Aβ_1-42_ antibody signals (Figure 3A-d, e, f). The co-localization patterns were further quantitatively presented by Plot Profile analysis, which displayed the fluorescence distribution profile of Aβ_1-42_ antibody signal (red line) and **AH-2** signal (green line) along line-x in Figure 3B-3D. It is noteworthy that the co-localization of the **AH-2** signal with the antibody signal was also observed in the hippocampus region of mice brain slices (Figure S12). These results suggest the desirable selectivity of **AH-2** towards Aβ deposits in biological conditions.

Secondly, the spatial resolution of **AH-2** imaging was investigated. Aβ deposits are commonly observed in the extracellular areas. However, recent studies addressed cellular Aβ deposits preceding extracellular Aβ plaques accumulation, being a key determining factor of neurological dysfunction in age-related neurodegenerative pathogenesis 36-40. To investigate the spatial distribution of **AH-2** stained aggregates, the aforementioned slices were co-stained with DAPI, a classic nuclear counterstain. It was observed that **AH-2** staining presented two different profiles of spatial distribution in both the 3xTg group and the 5xFAD group. Given that part of **AH-2** puncta covered several cell nuclei, these large size deposits should represent the extracellular Aβ plaques (Figure 3A and S7). In contrast, part of the **AH-2** positive deposits appeared as small intracellular puncta around the cell nucleus, representing the intracellular Aβ accumulation. Similar Aβ distribution profiles were verified in the brain slices stained with the Aβ_1-42_ antibody immunostaining (Figure 3A and S7). These results demonstrate the desirable spatial resolution of **AH-2** for staining both the intracellular and extracellular Aβ deposits in brain tissue.

Thirdly, we tested if **AH-2** would be sensitive enough to accurately stain Aβ deposits in the early stage of AD development or even before AD onset. For this purpose, aging wide-type mice at 2-, 8-, and 13-months were used, with age-matched 3xTg mice set as the positive control group. It was observed that **AH-2** signals enhanced in an age-dependent way in both the wide-type group and the 3xTg group (Figure 3E, 3G, and S8), suggesting the accumulation of Aβ aggregates in mice brains as the mice aged. Comparison of the wide-type and 3xTg AD groups revealed that the 3xTg AD mice demonstrated more Aβ deposits in both the cortex (Figure 3E) and the hippocampus region (Figure S13) than their wild-type counterparts at the same ages. Noteworthy, obvious Aβ deposits were observed in the cortex region of the wild-type group at 8 months old (Figure 3E), suggesting that **AH-2** was sensitive enough to image Aβ deposits in naturally aging wide-type mice. Interestingly, slices from the hippocampus region of the wild-type mice at 8 months old showed less obvious **AH-2** fluorescence (Figure S13), indicating that the cerebral cortex is more vulnerable to neurodegenerative stimulation during aging.

Spatially, **AH-2** imaging revealed that Aβ puncta signals appeared near the nuclei in young mice but spread through the intracellular and extracellular compartments in older mice in both wide-type and 3xTg mice. This is in agreement with previous reports that intracellular Aβ deposits precede extracellular ones during aging and AD development 36-40. The age-dependent accumulation of Aβ deposits in wild-type and 3xTg mice brain was also confirmed by **ThT** either in the cortex (Figure 3F and 3H) or hippocampus region (Figure S14). However, a comparison between **ThT** and **AH-2** imaging results showed that **AH-2** revealed significant statistical differences of Aβ puncta enhancement between the 3xTg group and the age-matched wide-type group (Figure 3G), which were not detectable using **ThT** staining in the present case (Figure 3H). These results further underscore the enhanced sensitivity of **AH-2** to stain Aβ deposits facilitating the prediction and diagnostic applications in AD.

### Testing the sensitivity of AH-2 in naturally aging live mice

**Figure 4:**
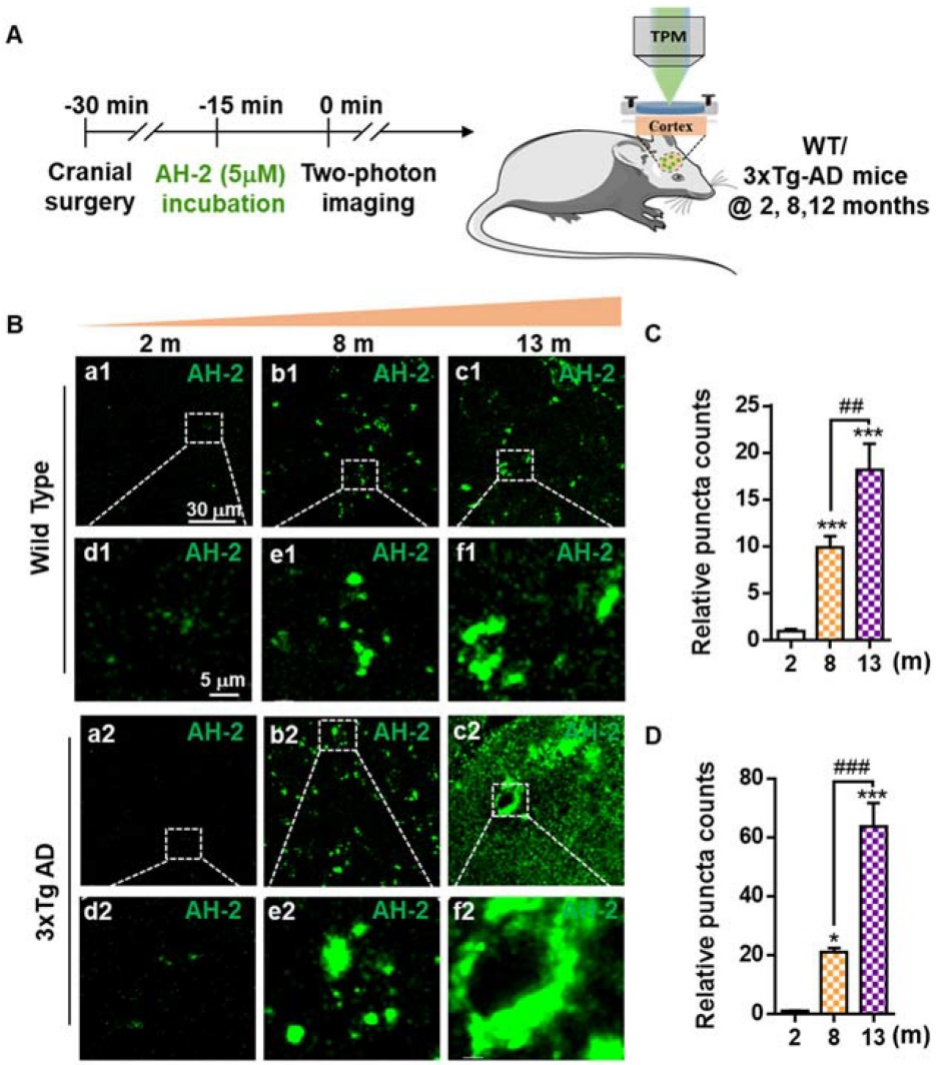
** Two-photon *in vivo* imaging of Aβ deposits in the brains of live mice. (A)** Schematic diagram of AH-2 application *in vivo* with two-photon imaging. **(B)** Images of AH-2 fluorescence-indicated Aβ deposits acquired from the brain cortex of aging wild-type mice and 3xTg AD mice. AH-2 signal was collected at 580 nm upon the two-photon excitation at 860 nm in the brains of live mice. Representative boxed regions were magnified and displayed in the bottom panel. **(C, D)** Quantitative analysis of AH-2 labeled Aβ puncta in wild-type and 3xTg AD mice at ages ranging from 2 months to 13 months. The data were expressed as Mean ± SEM. *p < 0.001, ***p < 0.001 versus 2 months group. ##*p* < 0.01, ###*p* < 0.001 compared to 8 months group. *N* = 5 mice.

Encouraged by the desirable selectivity and sensitivity of **AH-2** towards Aβ deposits in mice brain slices, we further investigated the feasibility of **AH-2** to stain native Aβ deposits in live mice. Two-photon laser scanning microscopy represents a desirable imaging modality to record *in situ* biological activity with deep tissue penetrations 27, 41, and was used for this study. The general procedures for this experiment was described in Figure 4A. Cranial imaging window surgery was carried out in anesthetized mice of both the wild-type and 3xTg mice. Then **AH-2** was administrated, followed by two-photon microscope imaging. Images of **AH-2** fluorescent signals in response to Aβ deposits in the cortex at 100 µm depth of wild-type mice and 3xTg AD mice were collected at 580 nm upon the two-photon excitation at 860 nm.

The imaging data showed that **AH-2** fluorescence signals increased extensively in the brains as the mice aged from 2-month to 13-month (Figure 4B-4D). For wild-type mice of 8- and 13-month-old, **AH-2** deposit counts increased dramatically in an age-dependent pattern when compared with the young mice of 2-month-old (Figure 4B and 4C), indicating the aging-related Aβ accumulation in the normal aging brain. Compared with the wild-type mice, the Aβ deposits increased more sharply in 3xTg AD mice aging from 2 months to 13 months (Figure 4D). These *in vivo* imaging results from live mice were consistent with those revealed by **AH-2** *ex vivo* imaging in brain slices (Figure 3), but with improved fidelity. These results provided further support for the hypothesis that Aβ aggregates happen in the naturally aging brains, and demonstrated the robustness of **AH-2** to stain Aβ aggregates *in vivo* with excellent sensitivity and spatial resolution.

### AH-2 imaging facilitated the efficacy evaluation of anti-AD agents

After confirming the desirable sensitivity and fidelity of **AH-2** to image Aβ deposits in naturally aging wide-type mice, we then tested if it would be applicable to evaluate the efficacy of anti-AD agents. The logic is that Aβ aggregation is not only a well-recognized AD diagnostic biomarker but also an indispensable prognostic indicator 42, 43. For this purpose, melatonin was selected as the positive agent because of its well-recognized efficacy against aging and AD 44-46. In this context, 3xTg AD mice (13-month) were treated with melatonin at a dose of 10 mg/kg/day for 6 weeks, and the amount of cerebral Aβ aggregates was monitored by **AH-2**. Noteworthy, the expression levels of cognitive proteins (Kinase C) were also assayed by western blot to confirm the neuro-protective efficacy of melatonin.

**Figure 5:**
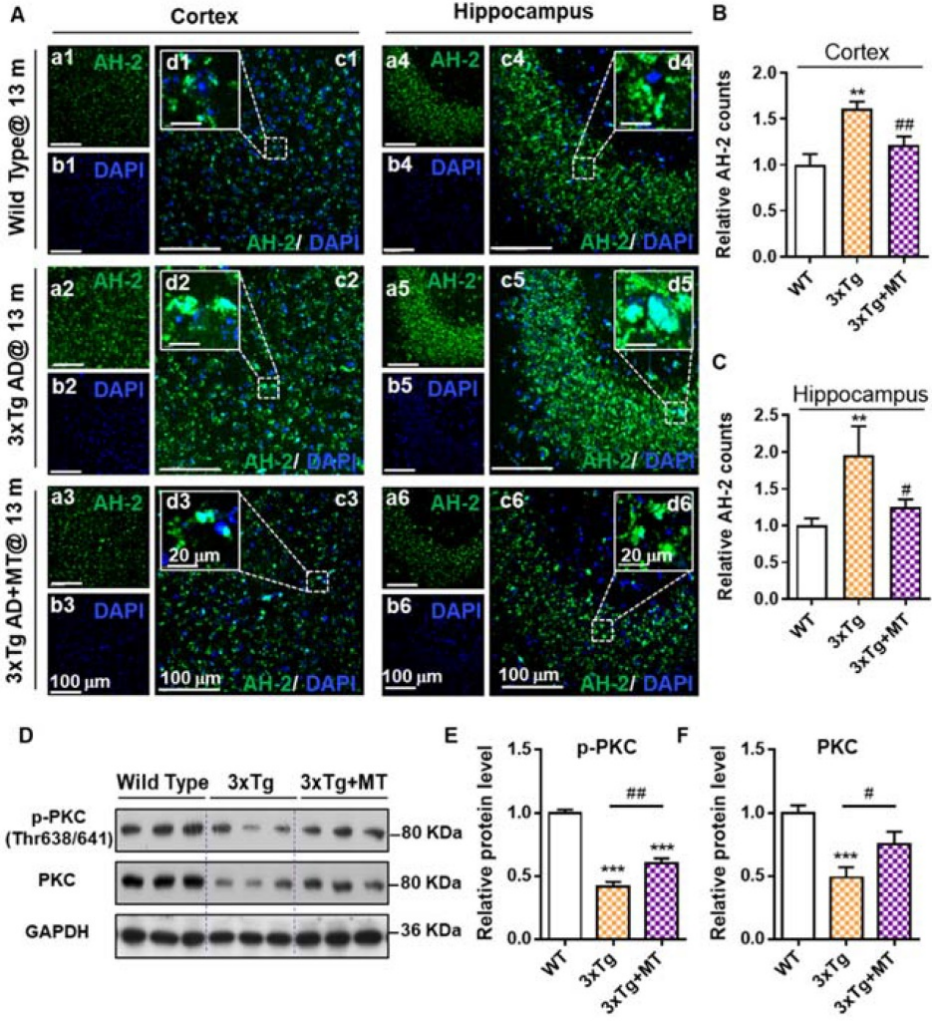
** AH-2 imaging revealed melatonin-induced reduction of Aβ deposits in AD mice brain. (A)** Representative confocal images of **AH-2** tracked Aβ aggregation changes in the cortex (left) and hippocampus (right) of aged wild-type mice (upper panel), 3xTg AD mice (middle panel), and melatonin-treated 3xTg AD mice (bottom panel). **AH-2** signal (green) was collected at 595 nm upon laser excitation at 488 nm. Magnified images (d1-d6) of white-box regions showed the expression patterns of Aβ aggregation around the DAPI (blue)-indicated nuclei. Scale bar = 100 µm (a-c). Scale bar = 20 µm (d).** (B, C)** Effect of melatonin on Aβ aggregation in the cortex (B) and hippocampus (C) was measured and quantified as the counts of **AH-2** positive puncta. All data were normalized to the wild-type group and presented as Mean ± SEM. **p < 0.01 versus wild type group. #p < 0.05, ##p < 0.01 versus 3xTg AD group. **(D)** Representative Western blot bands of cognition-related proteins *p*-PKC and PKC expression analysis in the hippocampus from wild type and 3xTg AD mice with and without melatonin treatment. GAPDH worked as sample load control for the blots. (E, F) Protein levels in each group were measured by Image J and shown as the ratio of the WT group. Data were expressed as Mean ± SEM. **p < 0.05, ***p < 0.001 versus WT group. #*p* < 0.05, ##*p* < 0.01 versus 3xTg group. *N* = 6 mice.

The images of **AH-2** staining showed that 3xTg AD mice (13 months) without melatonin treatment displayed a higher level of **AH-2** deposits in the cortex region and hippocampus when compared to the age-matched wild-type mice brain (Figure 5A-5C). After melatonin treatment, the **AH-2** puncta were significantly blunted both in size (Figure 5A-d3, d6) and in count (Figure 5B and 5C), revealing the benefit of melatonin treatment to decrease cerebral Aβ aggregates in AD mice. In addition, we also confirmed that melatonin treatment contributed to the neurological recovery in AD mice, as indicated by the recovery of cognitive-linked Protein Kinase C (PKC) which plays a crucial role in synaptogenesis and neuronal plasticity, and is needed for learning and memory 47-49. As shown in Figure 5D by Western blot analysis, comparing with the wild-type mice, 3xTg mice expressed significantly compromised PKC and phosphorylated PKC (p-PKC, the activated state of PKC) levels (Figure 5D-5F). Conversely, melatonin treatment remarkably restored the expression of both PKC and p-PKC in the hippocampus of 3xTg AD mice. These data suggested that melatonin reduced Aβ deposits effectively and protected the cerebral neurons from Aβ stress-induced injury; while probe **AH-2** was sensitive enough to image melatonin-rendered Aβ deposits reduction. We anticipate that **AH-2** should be promising to evaluate the efficacy of anti-AD agents.

## Conclusion

Aided by quantum chemical calculations, we have rationally designed probe **AH-2** sensitive enough to *in situ* image Aβ deposits in naturally-aging normal mice brains. The probe was a **ThT** derivative. It retained the binding affinity and binding mode of **ThT** towards Aβ deposits, as shown by the plot of its binding constants towards Aβ fibrils by the titration experiment. **AH-2** was designed to enhance the twisted intramolecular charge transfer (TICT) tendency of **ThT**, so as to reduce its background emissions. Due to its decreased background signals, **AH-2** sensitively stained Aβ aggregates with an improved signal-to-noise ratio by 5-10 times compared to **ThT**. This improved sensitivity enabled **AH-2** to detect a trace amount of Aβ deposits in wide-type mice before the onset of AD, which is more advantageous than those probes that can only be used in AD-modelled trans-genetic mice overexpressing Aβ deposits. We also showed that **AH-2** was active towards Aβ oligomers and can detect cellular Aβ deposits in mice brain tissues. Spatially, **AH-2** imaging revealed that Aβ first accumulated intracellularly and then spread extracellularly during the naturally aging process of wide-type mice; while the accumulation emerged earlier in the cortex region than in the hippocampus region during this process. Moreover, age-dependent Aβ accumulation in both wide type and AD-modelled trans-genetic mice was revealed by **AH-2** staining. We also showed that** AH-2** was sensitive enough to detect the reduction of Aβ deposits rendered by ani-AD agents, suggesting its potential application for the screening of anti-AD agents. Given the desirable sensitivity of **AH-2** and its desirable spatial resolution, we envision that the probe can be used as a robust tool in understanding the mechanism of native Aβ plaque formation, aiding the predictive and diagnostic prevention of AD, and facilitating the further development of Aβ-related therapies.

## Supplementary Material

Supplementary materials and methods, figures.
