# Supplementary material for: High-fidelity imaging of amyloid-beta deposits with an ultrasensitive fluorescent probe facilitates the early diagnosis and treatment of Alzheimer's Disease
Source: Theranostics. 2022 Feb 28;12(6):2549–59. doi: 10.7150/thno.68743 (PMC8965481; doi:10.7150/thno.68743)
Supplement: Supplementary file 1 — Supplementary materials and methods, figures. [file thnov12p2549s1.pdf]

Supplementary Material

High-fidelity imaging of amyloid-beta deposits with an ultrasensitive fluorescent probe facilitates the early diagnosis and treatment of Alzheimer's Disease

Rongrong Tao[1], Ning Wang[2], Tianruo Shen[3], Yuhang Tan[1], Yong Ren[2], Wenjing Wei[1], Meihua Liao[1], Davin Tan[3], Chunzhi Tang[1], Nenggui Xu[1], Huan Wang[4]*, Xiaogang Liu[3]*, Xin Li[2]*

[1] Medical College of Acupuncture-Moxibustion and Rehabilitation, Guangzhou University of Chinese Medicine, Guangzhou, Guangdong 510006, China

[2] College of Pharmaceutical Sciences, Zhejiang University, Hangzhou, Zhejiang 301158, China

[3] Fluorescence Research Group, Singapore University of Technology and Design, 8 Somapah Road, Singapore 487372, Singapore

[4] College of Life Science and Technology, Dalian University, Dalian, Liaoning 116622, China

* Corresponding authors: lixin81@zju.edu.cn, xiaogang_liu@sutd.edu.sg or 0012669@zju.edu.cn

Table of Contents

Materials and instruments for synthesis and structure characterization 3
Probe synthetic methods and characterization data 3
  Scheme S1. Synthesis of probes **AH-1** and **AH-2** 3
  Detailed synthetic procedures for **AH-1** and **AH-2** 3
  Scheme S2. Synthesis of probes **AH-3** and **AH-4** 5
  Detailed synthetic procedures for **AH-3** and **AH-4** 5
Computational Methods 6
Aβ oligomer and fibril preparation 7
Fluorescence property measurement 7
K_d measurement 8
Immunofluorescence 8
Protein level analysis by Western blot 9
Supplementary figures 10
  Figure S1. 10
  Figure S2. 11
  Figure S3 11
  Figure S4. 12
  Figure S5. 12
  Figure S6. 13

Figure S7 13
Figure S8 14
Figure S9 14
Figure S10. 15
Figure S11 15
Figure S12 16
Figure S13 17
Figure S14. 18
NMR traces of the probes 19
HRMS spectra traces of the probes 22
References 23

Materials and instruments for synthesis and structure characterization

All chemicals and reagents for probe synthesis were obtained from commercial suppliers at analytical grade and used without further purification. Reactions were monitored by thin-layer chromatography (TLC) carried out on 0.25 mm silica gel plates (60 F-254, Merker KGaA) using UV light as the visualizing agent. Compound purification was performed by flash column chromatrography over silica gel (40 – 63 μm particle size, Taiyang, Rushan). ^{1}H NMR spectra were recorded on a Bruker spectrometer (500 MHz) at 23 °C. ^{13}C NMR spectra were recorded on a Bruker spectrometer (126 MHz). NMR spectra were calibrated using residual undeuterated solvent as the internal references ($CDCl_3$, ^{1}H NMR = 7.26, ^{13}C NMR = 77.16). All chemical shifts were reported in parts per million (ppm) and coupling constants (*J*) in Hz. High-resolution mass spectra (HRMS) were measured on an Agilent 6224 TOF LC/MS spectrometer using ESI-TOF (electrospray ionization-time-of-flight).

Probe synthetic methods and characterization data

Scheme S1. Synthesis of probes AH-1 and AH-2

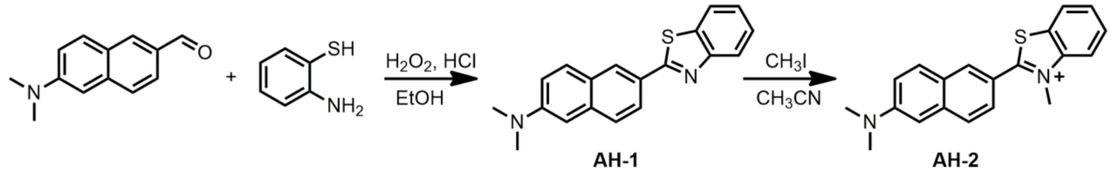

Detailed synthetic procedures for AH-1 and AH-2

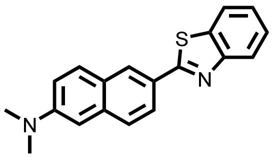

To an oven-dried flask was added 6-(dimethylamino)-2-naphthaldehyde (350 mg, 1.75 mmol) and 2-aminobenzenethiol (224 mg, 1.78 mmol). Then 80 mL of anhydrous EtOH was added to dissolve the solid. The flask was cooled with an ice bath. With vigorous stirring, concentrated hydrochloric acid (0.14 mL, 2.6 equivalent) and hydrogen peroxide (0.24 mL, 4.5 equivalent) were sequentially added to the solution. The mixture was stirred overnight at room temperature. TLC showed the complete disappearance of the starting materials. The mixture was then filtered. The filtrate was washed three times with anhydrous ethanol and dried to obtain **AH-1** as a yellow

solid (160 mg, 30% Yield). NMR trace showed that it was pure enough for direct application.

^{1}H NMR (500 MHz, $CDCl_3$) δ 8.42 (d, *J* = 1.3 Hz, 1H), 8.10 – 8.04 (m, 2H), 7.90 (d, *J* = 7.8 Hz, 1H), 7.82 (d, *J* = 9.1 Hz, 1H), 7.72 (d, *J* = 8.6 Hz, 1H), 7.48 (td, *J* = 9.1, 1.3 Hz 1H), 7.36 (td, *J* = 8.6, 1.3Hz 1H), 7.19 (dd, *J* = 8.6, 2.5 Hz, 1H), 6.92 (s, 1H), 3.10 (s, 6H).

^{13}C NMR (126 MHz, $CDCl_3$) δ 169.02, 154.46, 149.65, 136.65, 135.01, 130.02, 127.60, 127.14, 126.91, 126.32, 126.18, 125.048, 124.88, 122.92, 121.66, 116.79, 105.96, 40.74.

HRMS: $[M+H]^+$ calc'd for $C_{19}H_{17}N_2S$ 305.1112; found 305.1124.

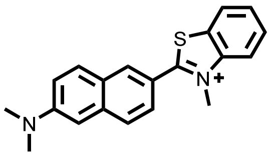

AH-1 (80 mg, 0.26 mmol) was dissolved in anhydrous acetonitrile (20 mL) in a thick-walled pressure bottle. Methyl iodide (0.16 mL, 10 equivalent) was added to the solution. The mixture was stirred at 80°C for 48 h. After evaporation of the volatile solvent, the crude product was purified first by column chromatography on silica gel (CH_2Cl_2-MeOH 20:1), followed by recrystallization with CH_2Cl_2 to give an orange solid (25 mg, 30% Yield).

^{1}H NMR (500 MHz, $CDCl_3$) δ 8.46 (s, 1H), 8.35 (d, *J* = 7.9 Hz, 1H), 8.13 (d, *J* = 8.4 Hz, 1H), 8.02 (d, *J* = 9.1 Hz, 1H), 7.77 (t, *J* = 7.8 Hz, 1H), 7.73 (d, *J* = 8.6 Hz, 1H), 7.69 (d, *J* = 8.5 Hz, 1H), 7.62 (t, *J* = 7.5 Hz, 1H), 7.17 (dd, *J* = 9.1, 2.3 Hz, 1H), 6.80 (s, 1H), 4.58 (s, 3H), 3.15 (s, 6H).

^{13}C NMR (126 MHz, $CDCl_3$) δ 174.03, 151.33, 142.79, 137.97, 133.06, 131.51, 130.16, 128.94, 128.56, 127.89, 125.84, 125.15, 124.41, 117.48, 117.29, 116.51, 104.77, 40.46, 40.33.

HRMS: $[M]^+$ calc'd for $C_{20}H_{19}N_2S$ 319.1263; found 319.1276.

Scheme S2. Synthesis of probes AH-3 and AH-4

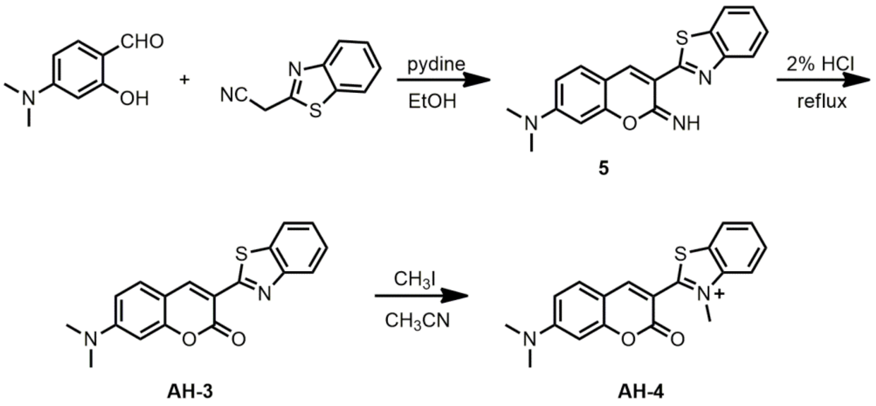

Detailed synthetic procedures for AH-3 and AH-4

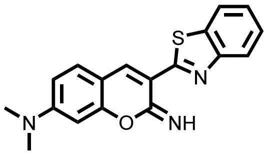

4-(Dimethylamino)-2-hydroxybenzaldehyde (200 mg, 1.21 mmol) and 2-(benzo[d]thiazol-2-yl)acetonitrile (211 mg, 1.21 mmol) was dissolved in anhydrous EtOH (10 mL). To this solution was added pyridine (0.1 equiv). The mixture was stirred at ambient temperature for 12 h. After that, the yellow precipitate was isolated by filtration. After a quick wash with H_2O, the solid was directly used for the next step (trace amount was kept for NMR analysis).

^{1}H NMR (500 MHz, $CDCl_3$) δ 8.27 (s, 1H), 8.03 (d, *J* = 8.1 Hz, 1H), 7.91 (d, *J* = 8.0 Hz, 1H), 7.49 (t, *J* = 7.7 Hz, 1H), 7.37 (t, *J* = 7.9 Hz, 1H), 7.34 (d, *J* = 8.7 Hz, 1H), 6.55 (dd, *J* = 8.7, 2.2 Hz, 1H), 6.46 (s, 1H), 3.09 (s, 6H).

^{13}C NMR (126 MHz, $CDCl_3$) δ 161.89, 161.14, 158.96, 156.82, 154.26, 142.40, 136.35, 130.73, 126.31, 124.71, 122.26, 121.77, 110.35, 109.17, 97.60, 40.42.

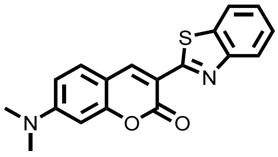

Intermediate **5** was treated with hydrochloric acid (2%, 10 mL). The mixture was refluxed for 10 h. TLC showed the complete disappearance of the starting material. The mixture was then cooled to room temperature, and neutralized with sodium acetate to pH 7-8. A solid is precipitated

which was filtered with suction. The solid was washed with water several times and dried in a desiccators to give **AH-3** as an orange solid (320 mg, 83% Yield for two steps).

^{1}H NMR (500 MHz, $CDCl_3$) δ 8.95 (s, 1H), 8.04 (d, *J* = 8.1 Hz, 1H), 7.95 (d, *J* = 7.9 Hz, 1H), 7.53 – 7.47 (m, 2H), 7.37 (t, *J* = 7.5 Hz, 1H), 6.70 (dd, *J* = 8.9, 2.4 Hz, 1H), 6.57 (d, *J* = 2.3 Hz, 1H), 3.12 (s, 6H).

^{13}C NMR (126 MHz, $CDCl_3$) δ 161.95, 161.11, 157.96, 156.86, 154.33, 142.54, 138.05, 130.80, 126.37, 124.76, 122.19, 121.79, 110.38, 109.19, 97.61, 40.44.

HRMS: $[M+H]^+$ calc'd for $C_{18}H_{15}N_2O_2S$ 323.0854; found 323.0848.

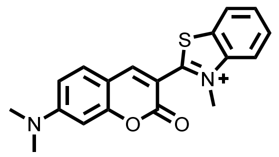

AH-3 (45 mg, 0.14 mmol) was dissolved in anhydrous acetonitrile (8 mL) in a thick-walled pressure bottle. Methyl iodide (0.088 ml, 10 equivalent) was added to this solution. The mixture was stirred at 80°C for 72 h. After evaporation of the volatile solvent, the mixture was purified by column chromatography on silica gel (CH_2Cl_2:MeOH 20:1) to yield **AH-4** as a red solid (35 mg, 74% Yield).

^{1}H NMR (500 MHz, $CDCl_3$) δ 8.94 (s, 1H), 8.03 (d, *J* = 8.3 Hz, 1H), 7.95 (d, *J* = 8.3 Hz, 1H), 7.53 – 7.45 (m, 2H), 7.37 (t, *J* = 7.0 Hz, 1H), 6.71 (dd, *J* = 8.9, 2.5 Hz, 1H), 6.59 (d, *J* = 2.5 Hz, 1H), 4.58 (s, 2H), 3.14 (s, 3H).

HRMS: $[M]^+$ calc'd for $C_{19}H_{17}N_2O_2S$ 337.1005; found 337.1020.

Computational Methods

Density functional theory (DFT),[1] and time-dependent density functional theory (TD-DFT) based theoretical calculations were conducted using *Gaussian 16.*[2, 3] All calculations were performed using ωB97XD functional and def2-SVP basis set in water.[4, 5] Positive values of frequencies were checked to validate the optimized structures in the ground and excited states. We accounted for solvent effects using SMD model with corrected linear-response (cLR) solvent formalism during soft scans of the potential energy surfaces.[6, 7] The molecular orbitals were visualized with *Avogadro* using the outputs from *Gaussian 16.*[8]

Aβ oligomer and fibril preparation

$A\beta_{1\text{-}42}$ (1 mg) was dissolved in 1 mL HFIP (Sigma Aldrich). The solution was sonicated for 10 min, aliquoted into 10 portions (each 0.1 mg), and dried in a vacuum. Each aliquot was stored in the refrigerator at -20ºC.

For oligomer preparation, one aliquot of the monomer was added with 11 μL DMSO, and then 432 μL PBS buffer (pH 7.4) to make a solution of 50 μM (calculated according to monomer amount). The sample was filtered with a 0.2 μm filter and then was kept at 4 ºC for 6 h.

For fibril preparation, one aliquot of the monomer was added with 11 μL DMSO, and then 432 μL PBS buffer (pH 7.4) to make a solution of 50 μM (calculated according to monomer amount). The solution was vortex spun for a few minutes, and then placed on a Vibrate for 24 h in a constant temperature box at 37°C. After cooling down to room temperature, the solution was placed in an icebox, and fibril formation was confirmed using electron microscopy (PHILIPS TECNAI 10).

Fluorescence property measurement

Fluorescence spectra were recorded on an Agilent Cary Eclipse Fluorescence Spectrophotometer. UV-Vis spectra were recorded on a Hitachi U-3010 spectrophotometer.

All the experiments were carried out at an ambient temperature in phosphate buffer saline (PBS, pH 7.4, from Solarbio). The probes were first dissolved in DMSO (Sigma Aldrich) to make 5 mM stock solutions. Then aliquots of the probe solution were diluted with PBS to the desired concentration, to which a solution of Aβ fibrils in PBS was added to the desired final concentration. After standing at ambient temperature for 5 min, the spectra were collected at specific absorption wavelengths.

For selectivity measurement, aliquots of **AH-2** stock solution were diluted with PBS to a final concentration of 2 μM, and then treated with various analytes at indicated final concentration. After 5 min of incubation, the fluorescence spectra were collected at excitation of 491 nm. Then the fluorescence intensity at 587 nm was plotted against the analytes.

K_d measurement

For k_d measurement between probe **AH-2** and Aβ fibrils, the experiment was carried out on a microplate reader (Biotek Synergy H1). The microplate reader was kept at λ_{ex} = 491 nm, λ_{em} = 491-700 nm. Aβ fibril stock solution in PBS (50 μM) was prepared as above mentioned. **AH-2** was dissolved in DMSO to make a stock solution of 50 μM.

K_{d1} measurement (Aβ titrated to **AH-2**). Record 200 μL PBS emission spectrum (blank). To the above PBS solution, add 0.4 μL **AH-2** (final **AH-2** conc. 100 nM) and record the emission spectrum of the above solution. Then to the **AH-2** solution, add Aβ fibrils in the portion (each portion 0.4 μL which corresponded to a final Aβ Conc. increase 100 nM). The mixture was stirred after each portion addition and was recorded for the emission spectrum. Read the fluorescent intensity (FI) at 575 nm. Three replicates were performed for the experiment. Calculate K_{d1} with Origin software (non-linear regression, Logistic).

K_{d2} measurement (**AH-2** titrated to Aβ). Record 200 μL PBS emission spectrum (blank). To the above PBS solution, add 0.4 μL Aβ (final Aβ conc. 100 nM) and record the emission spectrum of the above solution. Then to the Aβ solution, add **AH-2** in portions (each portion 0.4 μL which corresponded to a final **AH-2** Conc. increase 100 nM). Stir the mixture after each portion addition and record the emission spectrum. Read the fluorescent intensity (FI) at 575 nm. Three replicates were performed for the experiment. Calculate K_{d2} with Origin software (non-linear regression, Logistic).

Immunofluorescence

Mice were anesthetized with isoflurane and then perfused with PBS and subsequent 4% paraformaldehyde. The brain samples were isolated and fixed in 4% PFA at 4°C overnight. After fixation, samples were transferred to 30% sucrose solution and kept at 4°C for 24 h. Brain tissue slices with 40 μm thickness were prepared with cryostat sectioning followed with Aβ aggregation probe **AH-2** staining, **ThT** staining, and immunostaining. For probe staining, **AH-2** was dissolved in DMSO as a stock solution of 20 mM and diluted to 5 μM. **ThT** solution was prepared with water at 10 mM as stock solution and handled at 5 μM. Brain slices were washed with PBS three times and then incubated with probe **AH-2** or **ThT** for 10 minutes at room temperature. Following incubation and staining, slices were washed and shake in PBS for 30 minutes. To confirm the

AH-2 specificity to Aβ aggregation, co-staining of Aβ antibody and **AH-2** was performed. Briefly, brain tissue slices were treated with 0.1% triton x-100 for 15 min and then incubated in blocking buffer for 1 h at room temperature. Samples were incubated with primary antibody to Aβ (1:500, NBP2-13075, Novus Biologicals) for 24 h at 4°C and labeled with Alexa fluor 647-secondary antibody (1:500, A-31571, Thermo Fisher Scientific, Massachusetts, US), followed by **AH-2** staining as described above. Finally, slices were mounted with the antifade medium containing DAPI (H1200, Vector Labs, California, US). The fluorescence images and z-stack images were acquired by Nikon A1R confocal laser scanning microscope. **AH-2,** λ_{ex} = 488 nm, λ_{em} = 595 nm; **ThT**, λ_{ex} = 488 nm, λ_{em} = 520 nm; Aβ antibody, λ_{ex} = 640 nm, λ_{em} = 700 nm; DAPI, λ_{ex} = 405 nm, λ_{em} = 450 nm. Imaris and Image J were used for 3D-reconstructed images and image analysis.

Protein level analysis by Western blot

The brains of deeply anesthetized mice were quickly removed and dissected out. The protein extracts were performed on ice with lysis buffer containing cocktail protease inhibitors (Sigma, P8340). The protein sample concentration from each lysate was determined using Bradford's solution kit (P0006, Beyotime, Shanghai, China). Equal amounts of protein were loaded and separated by sodium dodecyl sulfate-polyacrylamide (SDS-PAGE) gel electrophoresis and then transferred to PVDF membrane for 1 h at 200 mA. Membranes were blocked in 5% fat-free milk in Tris-buffered saline containing 0.05% Tween-20 (TBST). Proteins were detected by specific primary antibodies against PKC (2056S, Cell Signal Technology, Massachusetts, US), p-PKC (9375S, Cell Signal Technology, Massachusetts, US), and GAPDH (70-Mab5465-040, Multi-Sciences Biotech, Hangzhou, China). All blots were incubated with the primary antibodies in blocking buffer overnight at 4°C. After incubation and rinsing at least three times in TBST, the membranes were incubated with HRP-linked secondary antibodies (7074S, 7076S, Cell Signal Technology, Massachusetts, US). The bands were visualized by enhanced chemiluminescence (34580, Thermo Fisher Scientific, Massachusetts, US) and quantified using Image J.

Supplementary figures

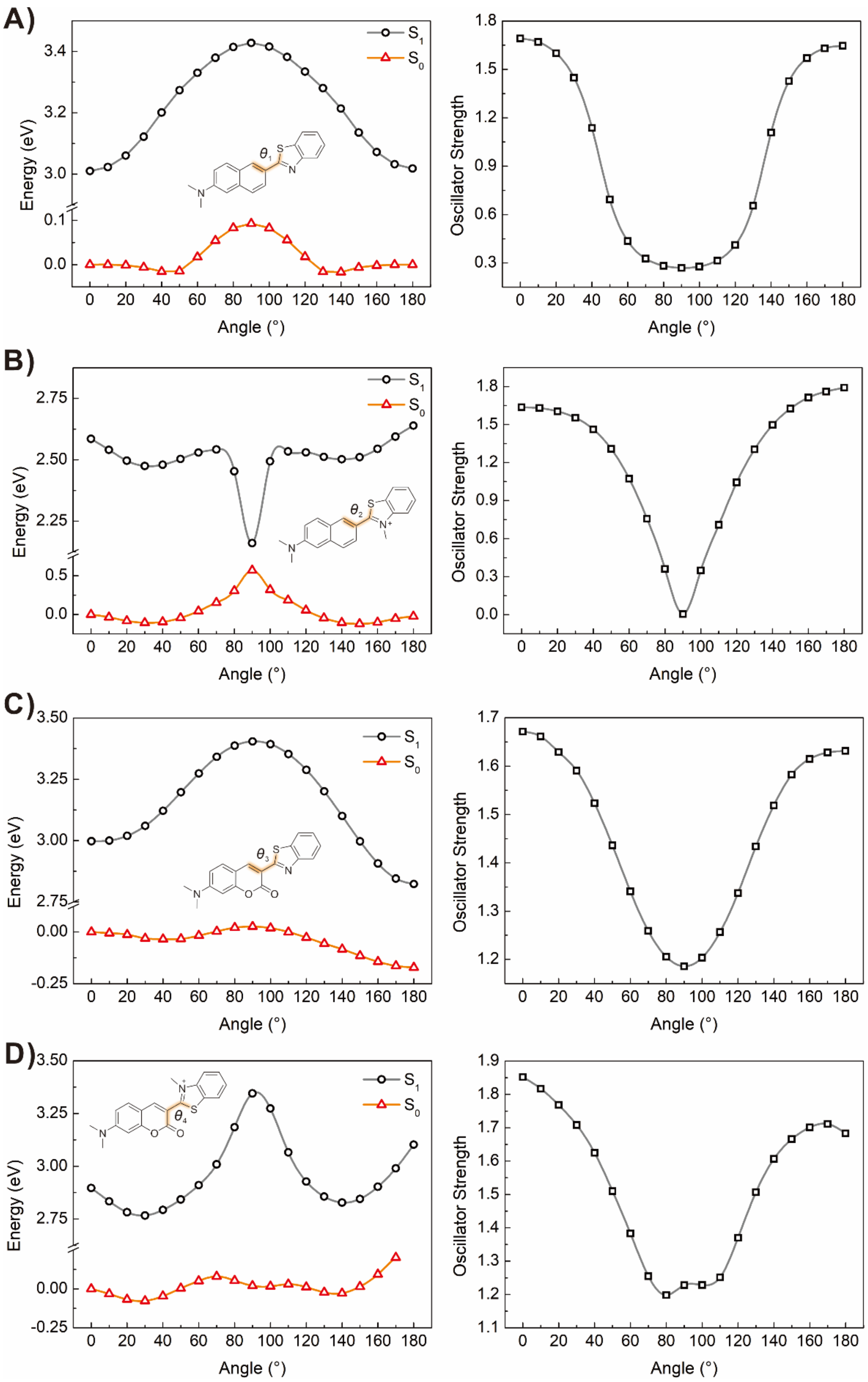

Figure S1. Calculated potential energy surface (left) and oscillator strength (right) of (A) AH-1, (B) AH-2, (C) AH-3, and (D) AH-4 as a function of θ_x in water.

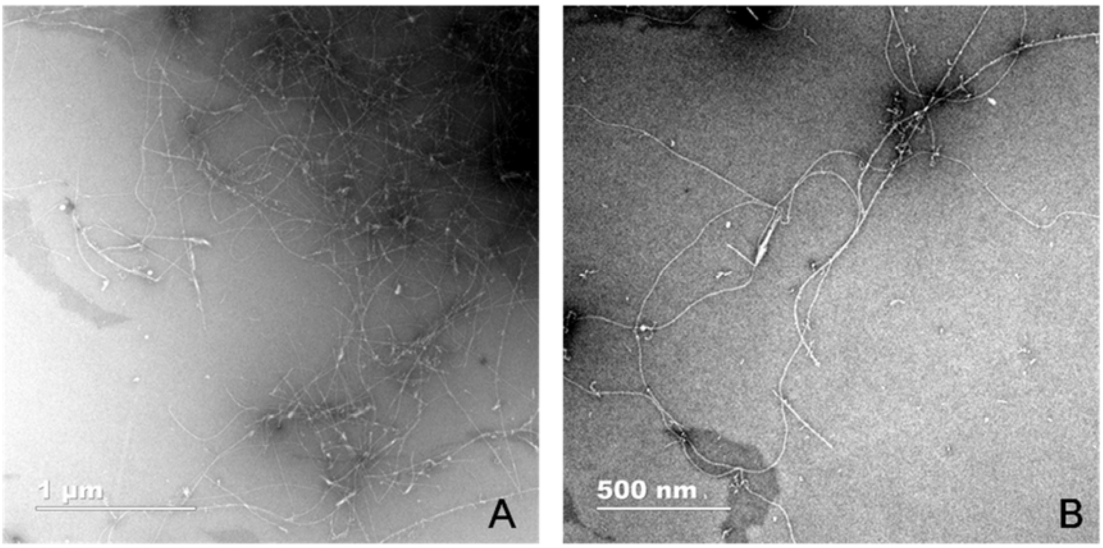

Figure S2. TEM images of Aβ (1-42) fibril. Aβ fibril was prepared as afore-mentioned. And fibril formation was recorded on a transmission electron microscopy (PHILIPS TECNAI 10) . A) scale 1 μm. B) scale 500 nm.

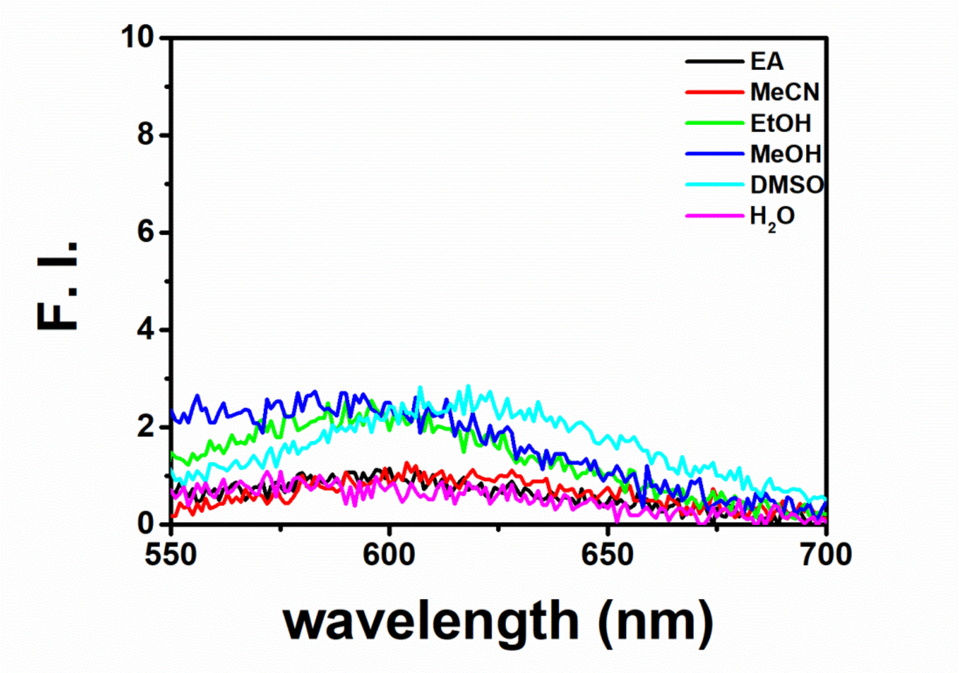

Figure S3. Fluorescence spectra of AH-2 in solvents of various polarities. AH-2 was kept at 5 μM, and excitation was conducted at the maximum absorption wavelength. Spectra were recorded on the Cary Eclipse Fluorescence Spectrometer.

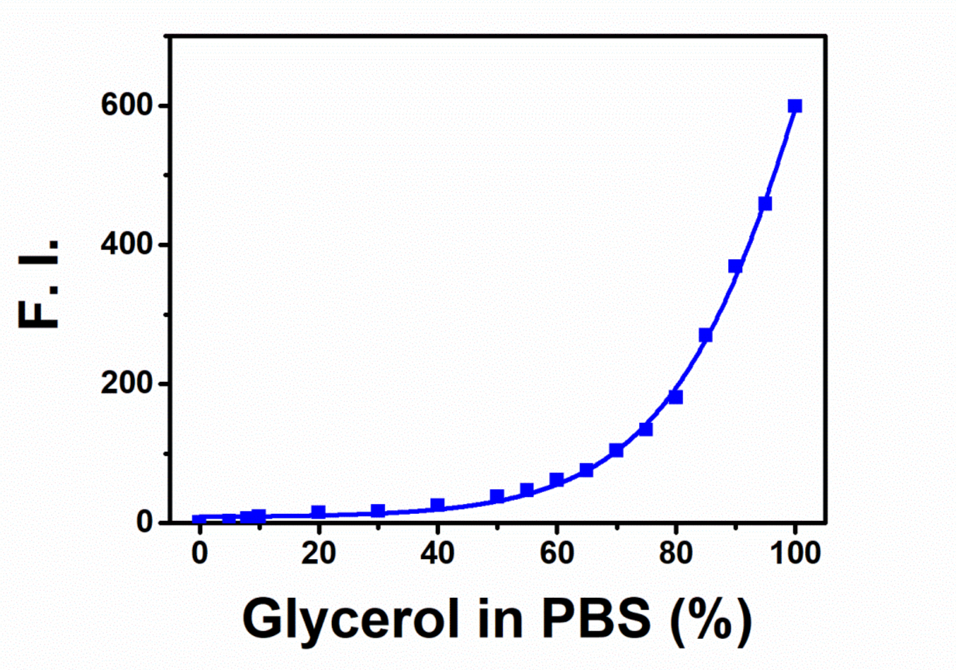

Figure S4. Plot of AH-2 fluorescence intensity at 600 nm versus the glycerol volume percentage of the solution. AH-2 (5 μM) in PBS solution containing various amounts of glycerol was recorded for its emission spectra on a Cary Eclipse Fluorescence Spectrometer (λ_{ex} = 450 nm). And the emission intensity at 600 nm was plotted against the glycerol volume percentage of the solution.

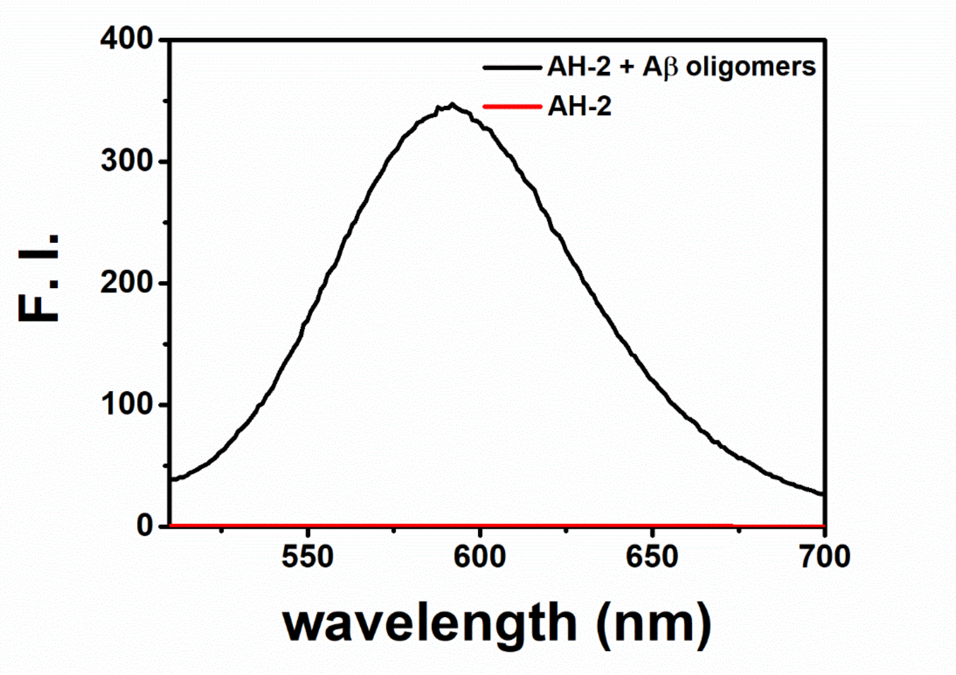

Figure S5. Fluorescence spectra of AH-2 (5 μM) before or after the treatment of Aβ oligomers (10 μM).

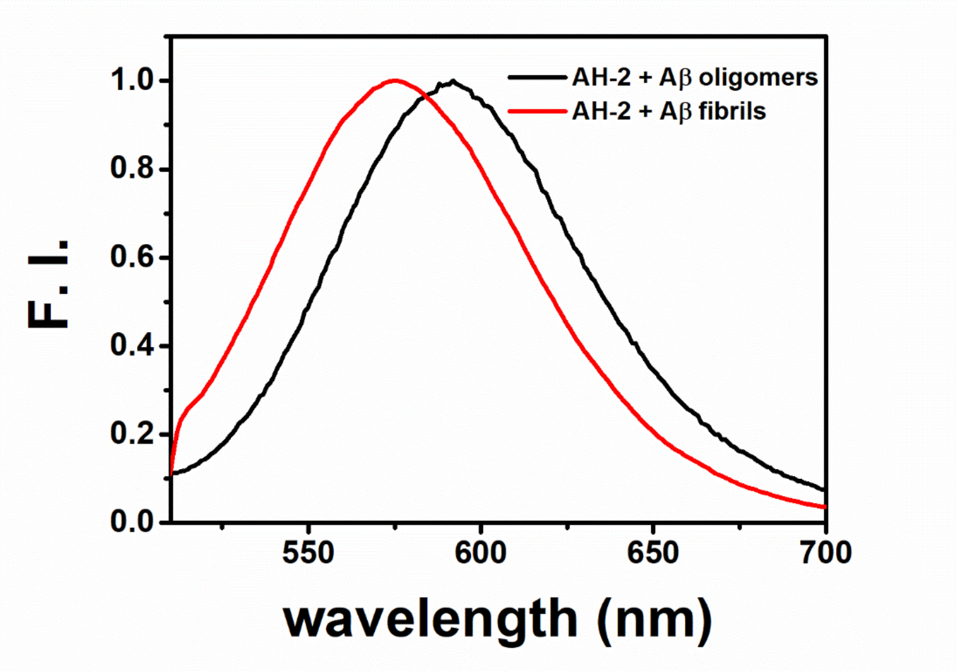

Figure S6. Normalized emission spectra of AH-2 in response to Aβ oligomers and Aβ fibrils.

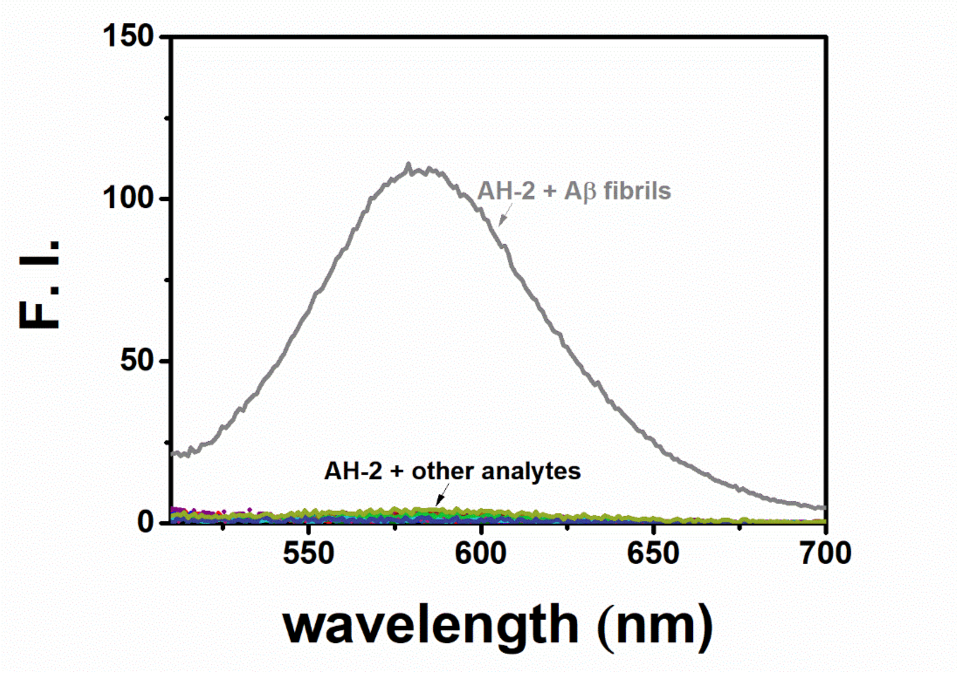

Figure S7. Fluorescence spectra of AH-2 (2 μM) after the treatment of various bio-relevant analyte. The tested analytes included 1) probe blank; 2) Na^{+}; 3) K^{+}; 4) Ca^{2+}; 5) Mg^{2+}; 6) Zn^{2+}; 7) Cu^{2+}; 8) Mn^{2+}; 9) Al^{3+}; 10) Fe^{3+}; 11) Arg; 12) Glu; 13) His; 14) Leu; 15) Hcy; 16) Cys; 17) GSH; 18) carboxyl esterase (1U/mL); 19) Aβ fibrils (10 μM). All species were kept at 200 μM except otherwise stated. Spectra were recorded on a Cary Eclipse Fluorescence Spectrometer. λ_{ex} = 491 nm.

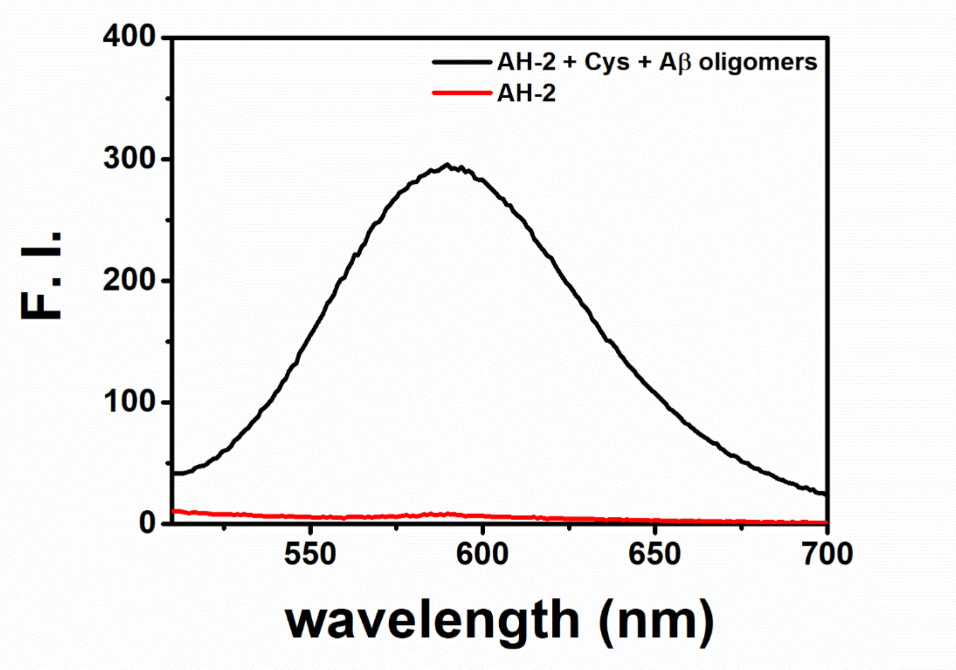

Figure S8. Fluorescence spectra of AH-2 after the subsequent treatment of Cys and Aβ oligomers. AH-2 (5 μM) in PBS solution was first treated with Cys (200 μM) for 5 min. Then the solution was further treated with Aβ oligomers (10 μM) and incubated for another 20 min. Finally the fluorescence spectra was colleted on a Cary Eclipse Fluorescence Spectrometer (λ_{ex} = 491 nm).

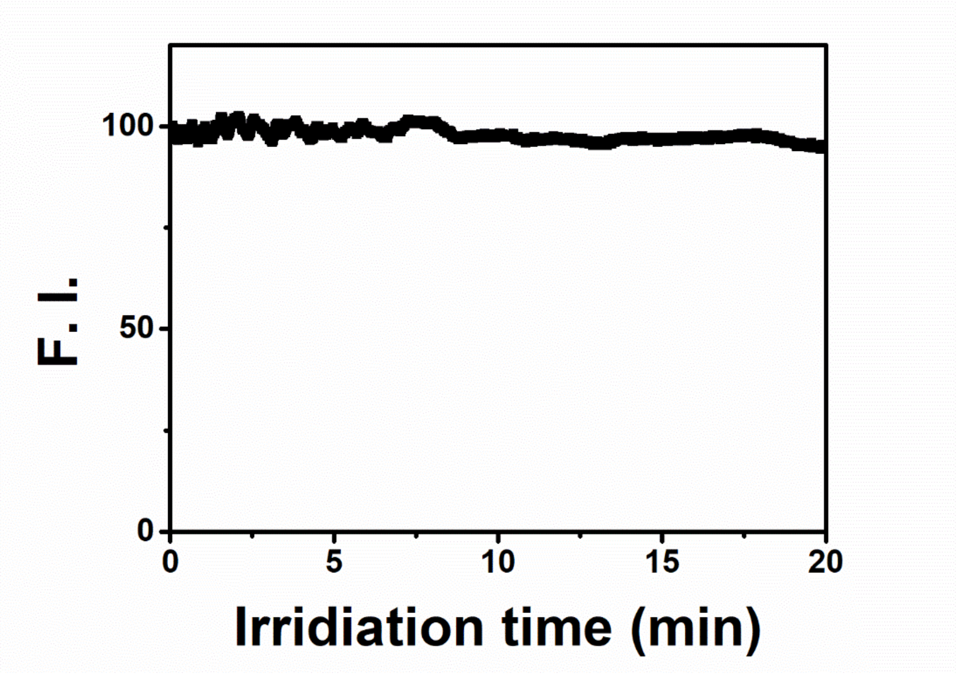

Figure S9. Photostability of AH-2 in response to Aβ fibrils. A solution of **AH-2** (2 μM) in PBS was treated with Aβ fibrils (10 μM) and incubated for 20 min. Then the solution was placed on a Cary Eclipse Fluorescence Spectrometer and the emission intensity at 580 nm was collected under the continuous excitation (λ_{ex} = 491 nm).

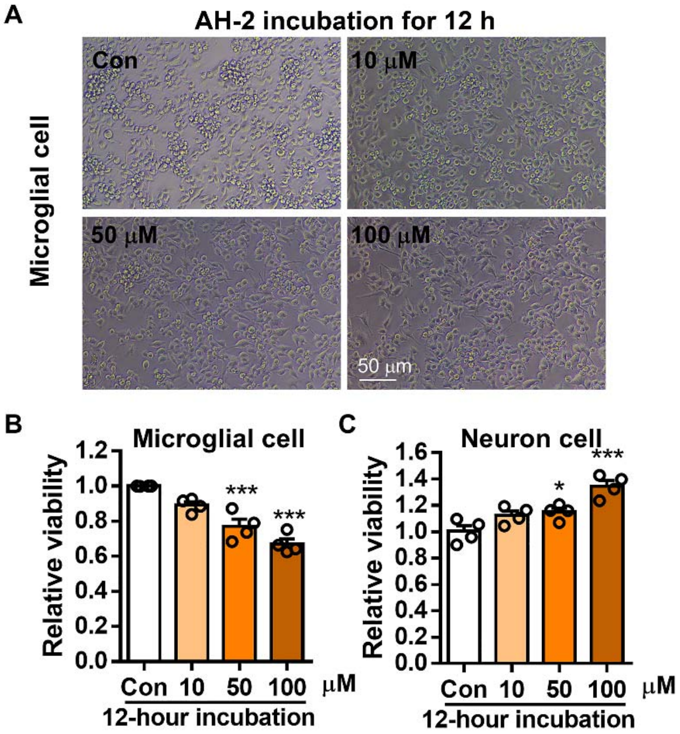

Figure S10. The cytotoxicity of probe AH-2 was evaluated in microglial cells and neuronal cells by cell viability analysis. (A) Representative images of microglial cell BV2 cultures following AH-2 incubation for 12 hours. Cell death was not observed after 12 hours AH-2 incubation except for mildly suppressed proliferation. (B) Quantification of microglial cell (BV2) viability after AH-2 incubation with cell counting kit (CCK-8) analysis. (C) Quantification of neuron cell (HT22) viability after AH-2 incubation with CCK-8 analysis. All data were presented as Mean ± SEM. **p* < 0.05, ****p* < 0.001 versus control group. n=4 trials.

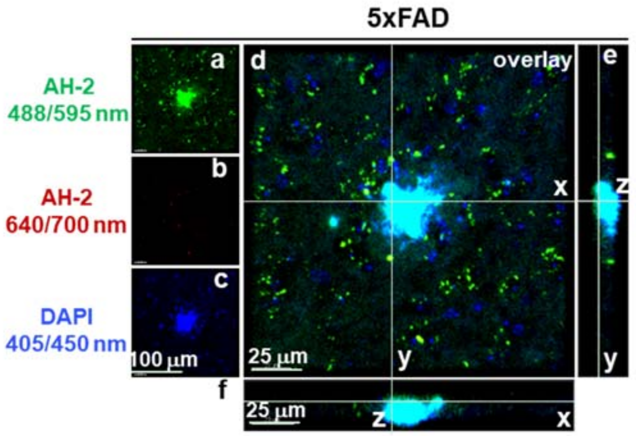

Figure S11. Evaluation of AH-2 bleed-through fluorescence signal upon excitation at 647 nm. Representative images of **AH-2** fluorescence signal responding to Aβ deposits in 6 months old

5xFAD mice, which were respectively captured at 595 nm with λ_{ex} = 488 nm (a, green channel) and 700 nm with λ_{ex} = 640 nm (b, red channel) using a confocal laser microscope. DAPI was used to stain the nuclei (c, blue channel, λ_{ex} = 405 nm, λ_{em} = 450 nm). The Z-stack image (d) and projection to z-y (e) and z-x (f) displayed the **AH-2** signal from the green channel rather than from the red channel, suggesting that no fluorescence signal from **AH-2** bleed through to the red channel upon excitation at 647nm. Scale bar 100 µm (a-c). Scale bar 25 µm (d-f).

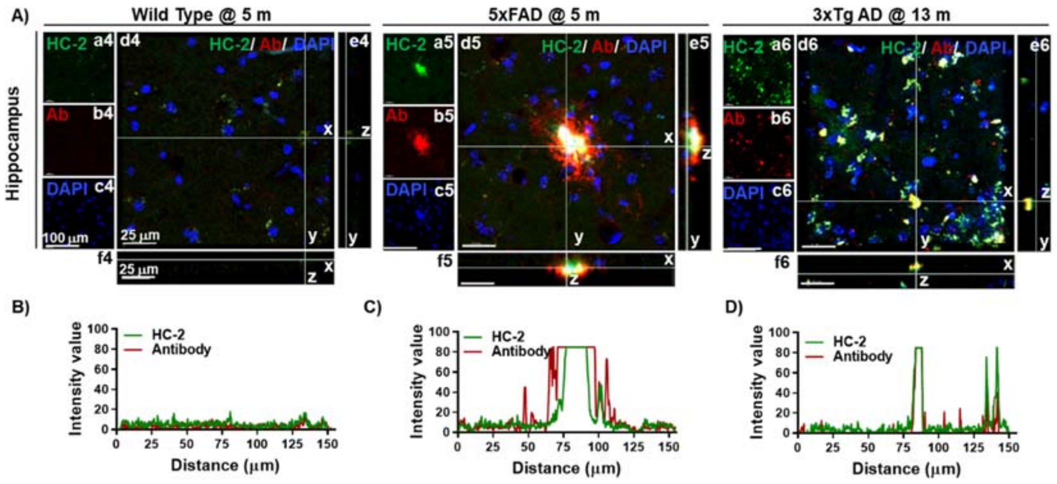

Figure S12. Staining Aβ deposits with AH-2 in mice brain slices from hippocampus. (A) Representative confocal images of Aβ deposits staining in the brain hippocampus of wild-type mice, 5xFAD mice, and 3xTg AD mice. Aβ deposits were co-stained by probe **AH-2** of 5 µM (green channel, λ_{ex} = 488 nm, λ_{em} = 595 nm) and $A\beta_{1-42/1-40}$ antibody at 1:500 dilution (red channel, λ_{ex} = 640 nm, λ_{em} = 700 nm). Cell nuclei were indicated by DAPI staining (blue channel, λ_{ex} = 405 nm, λ_{em} = 450 nm). The orthogonal projections of Z-stack images (d4-d6) to y-z (e4-e6) and x-z (f4-f6) further demonstrated the co-localization of **AH-2** signal and Aβ antibody signal, corroborating the selectivity of **AH-2** in detecting Aβ aggregates. **(B-D)** The fluorescence distributions of Aβ antibody signal (red line) and **AH-2** signal (green line) along line x in d4-d6 were analyzed with Plot Profile in Image J. The x-axis and y-axis represent the distance along line x in d4-d6 and fluorescence intensity value, respectively. N = 5 in each mice group.

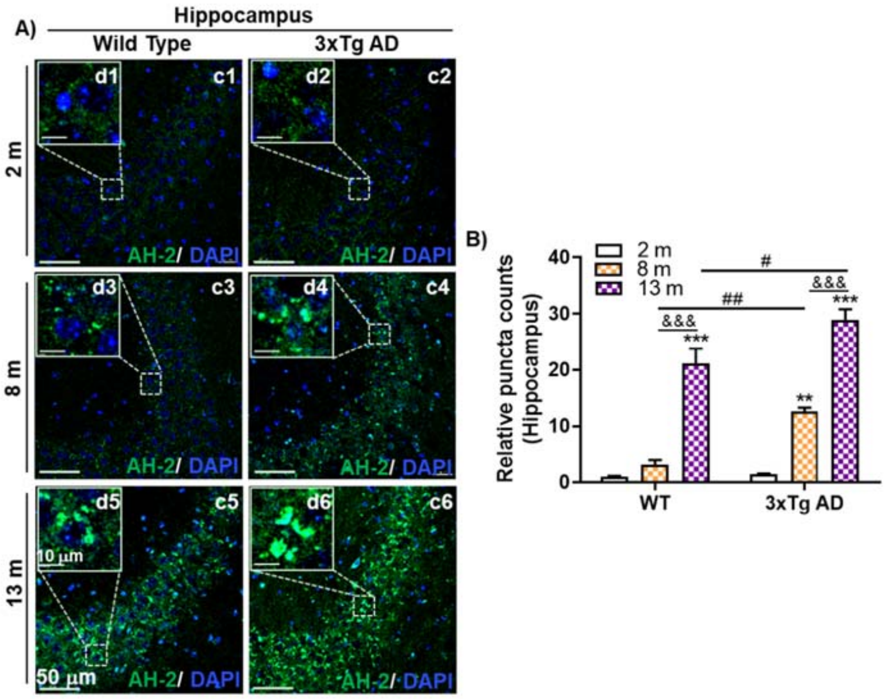

Figure S13. Staining Aβ deposits with AH-2 in naturally aging mice brain slices from the hippocampus region. **(A)** Confocal images of **AH-2** staining the age-dependent Aβ accumulation in the wild-type mice and 3xTg mice at ages of 2, 8, and 13 months. Magnified images of boxed regions were displayed in images d1-d6 respectively. **(B)** Quantification of **AH-2** labeled Aβ deposits in A. Data were expressed as Mean ± SEM normalized to WT at 2 months. **p < 0.01, ***p < 0.001 versus WT-2 months group. *&&& p* < 0.001 versus 8 months group. #*p* < 0.05, ##*p* < 0.01 versus age-matched WT group. *N* = 5 mice. Scale bar 50 µm.

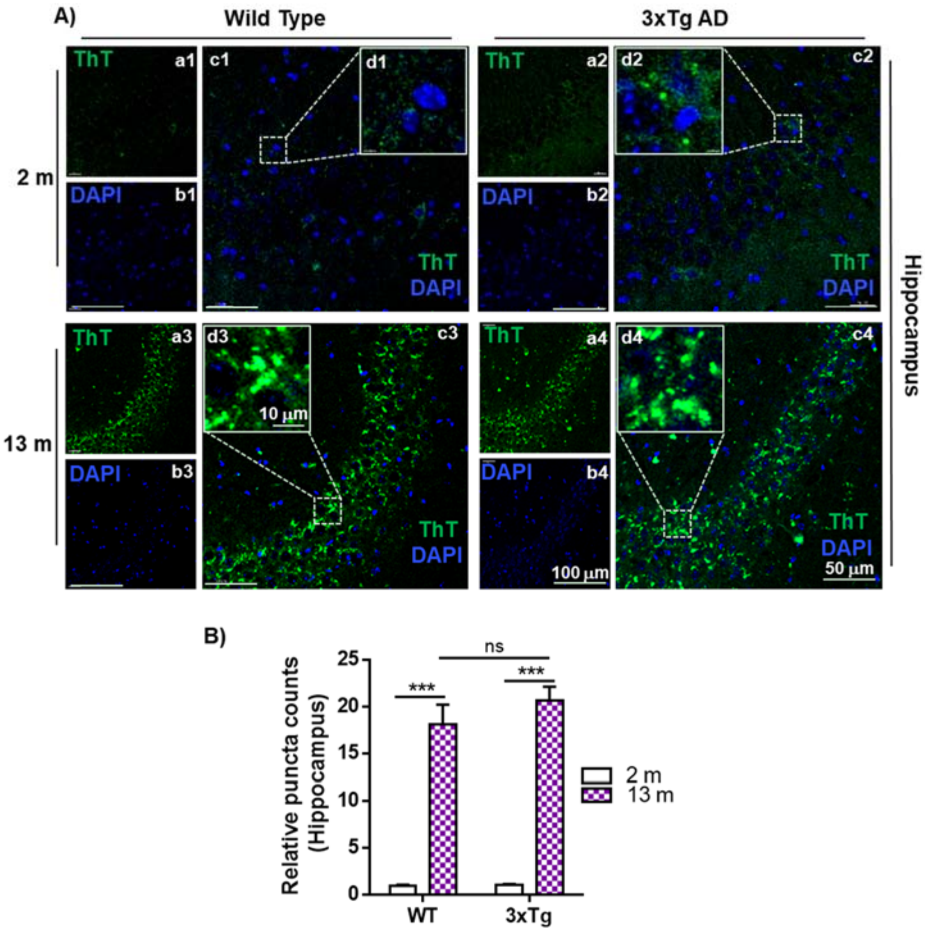

Figure S14. ThT staining of Aβ deposits in the naturally aging mice brain slices from the hippocampus region. (A) Representative images of **ThT** fluorescence tracking Aβ aggregation in the hippocampus of mice brain slices from wild-type mice and 3xTg AD model mice at ages of 2, 8, 13 months. Green fluorescence signal observed in 2 months wild-type mice was dim as shown in Panel a1 and c1, as well as the **ThT** signal in young 3xTg mice (a2 and c2). **ThT** signal was greatly increased in aged mice both from the wild-type group (a3 and c3) and 3xTg group (a4 and c4). DAPI (blue) indicated nuclei. The **ThT** and DAPI overlay images in Panel d1-d4 showed magnification of white boxed regions in Panel c1-c4 from wild type and 3xTg AD model mice respectively. **(B) ThT**-indicated Aβ aggregation was quantified and plotted as the puncta counts that normalized to 2 months WT group. No statistical difference was observed in the 3xTg AD group compared to WT group at age of 13 months. The data were expressed as Mean ± SEM. ***p < 0.001 versus 2 months group. ns, no significance. $N = 5$ mice. Scale bar 100 μm (a, b). Scale bar 50 μm (c). Scale bar 10 μm (d).

NMR traces of the probes

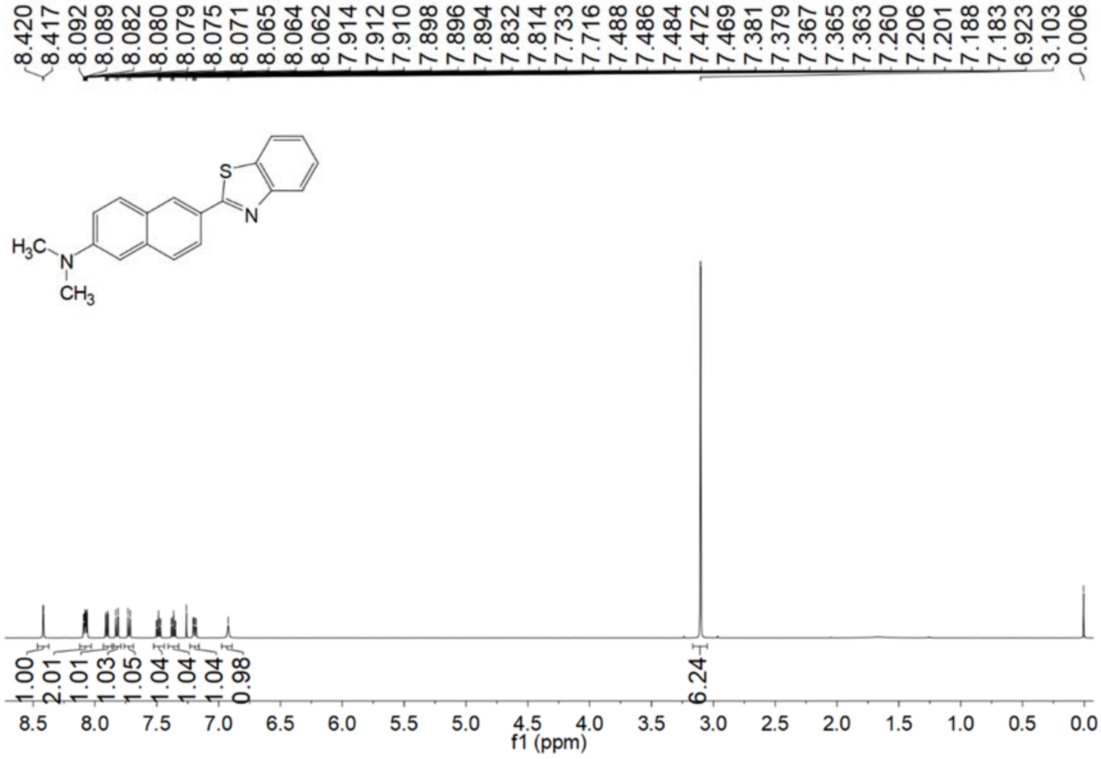

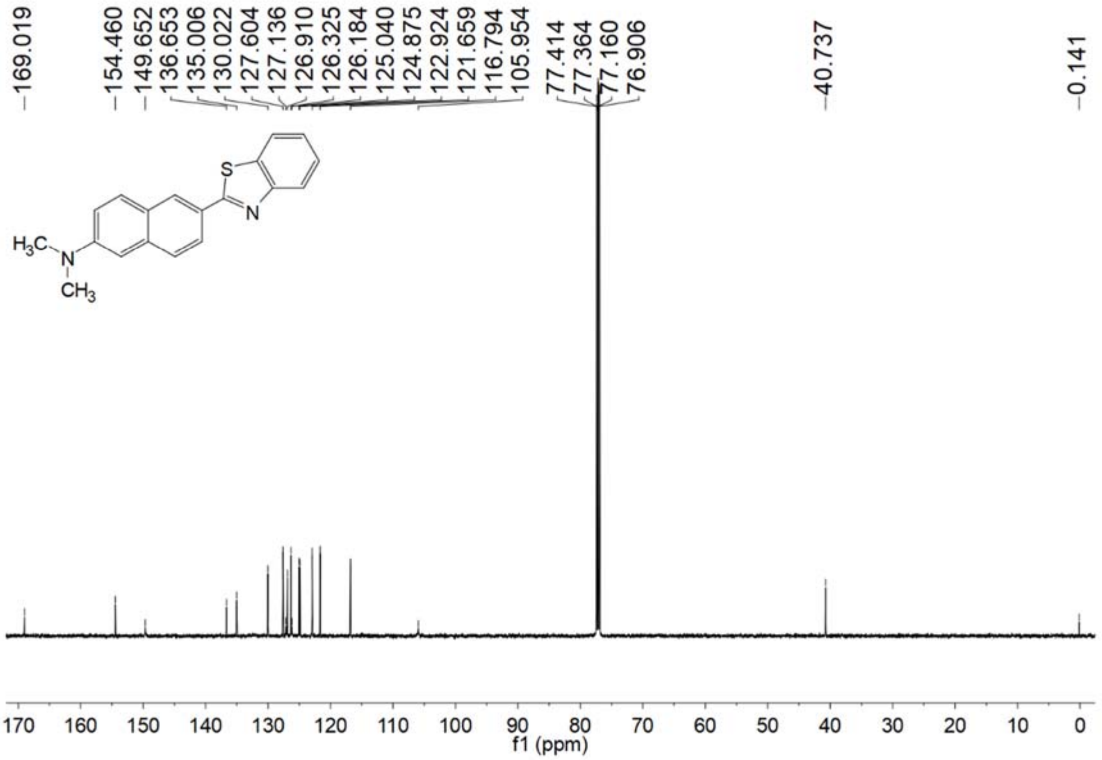

^1H and ^{13}C NMR of AH-1

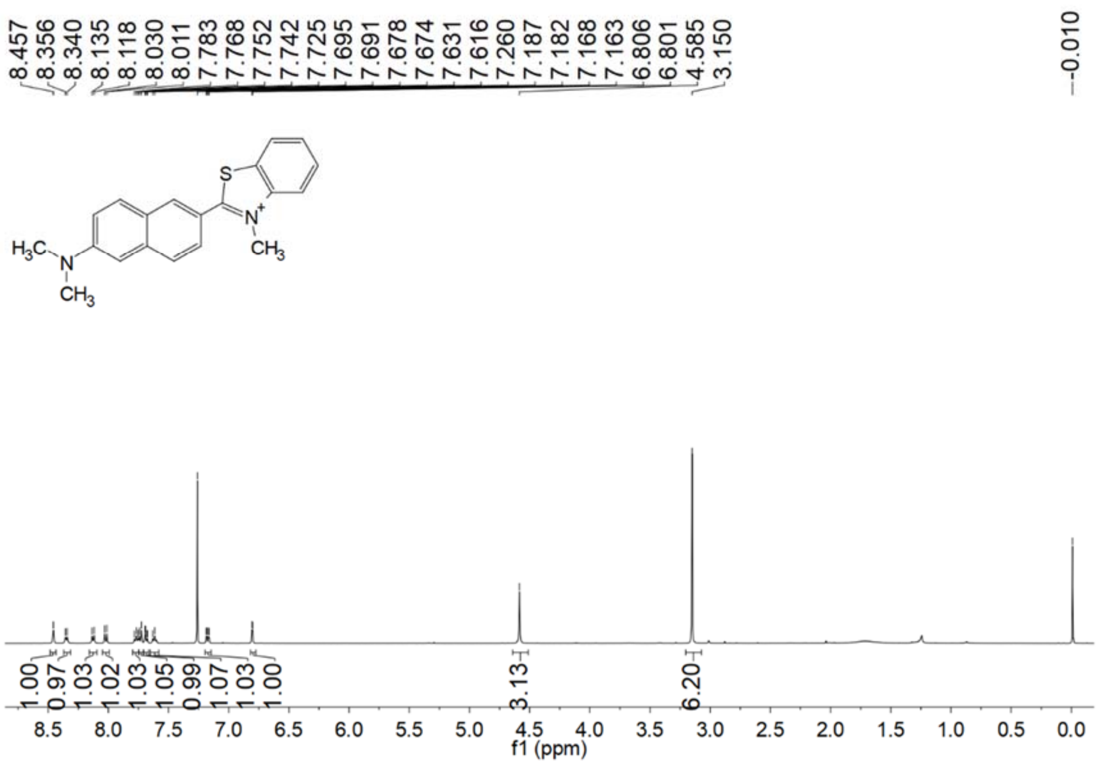

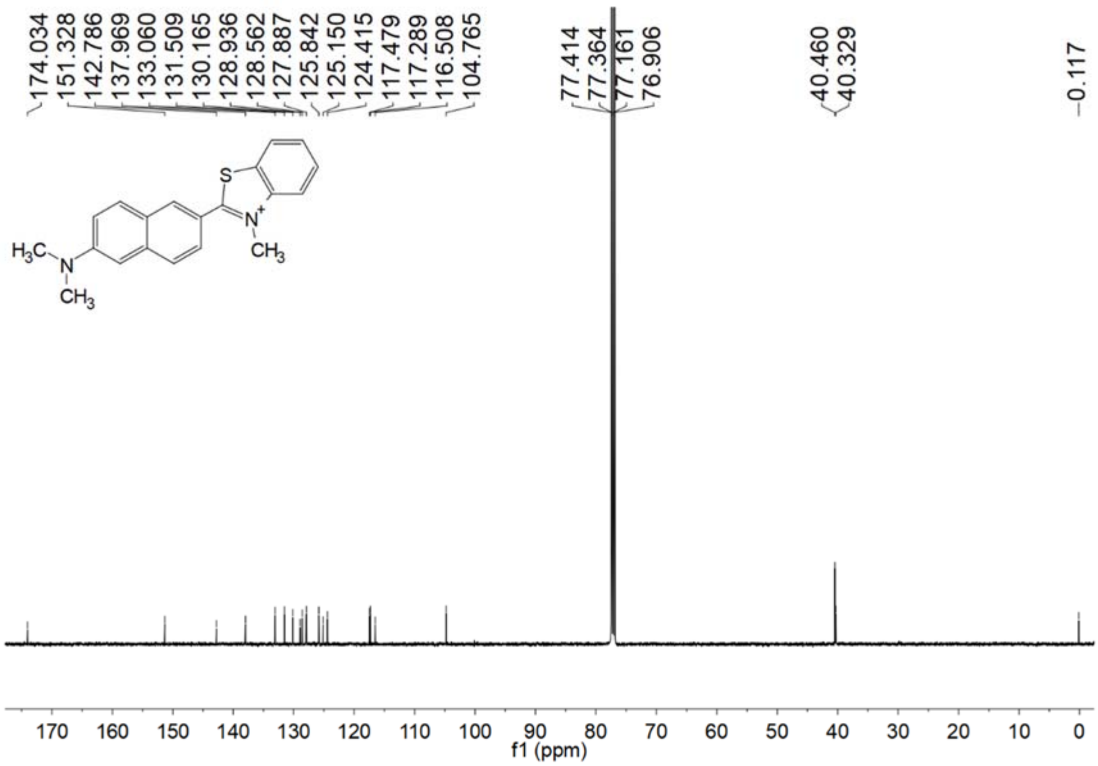

^{1}H and ^{13}C NMR of AH-2

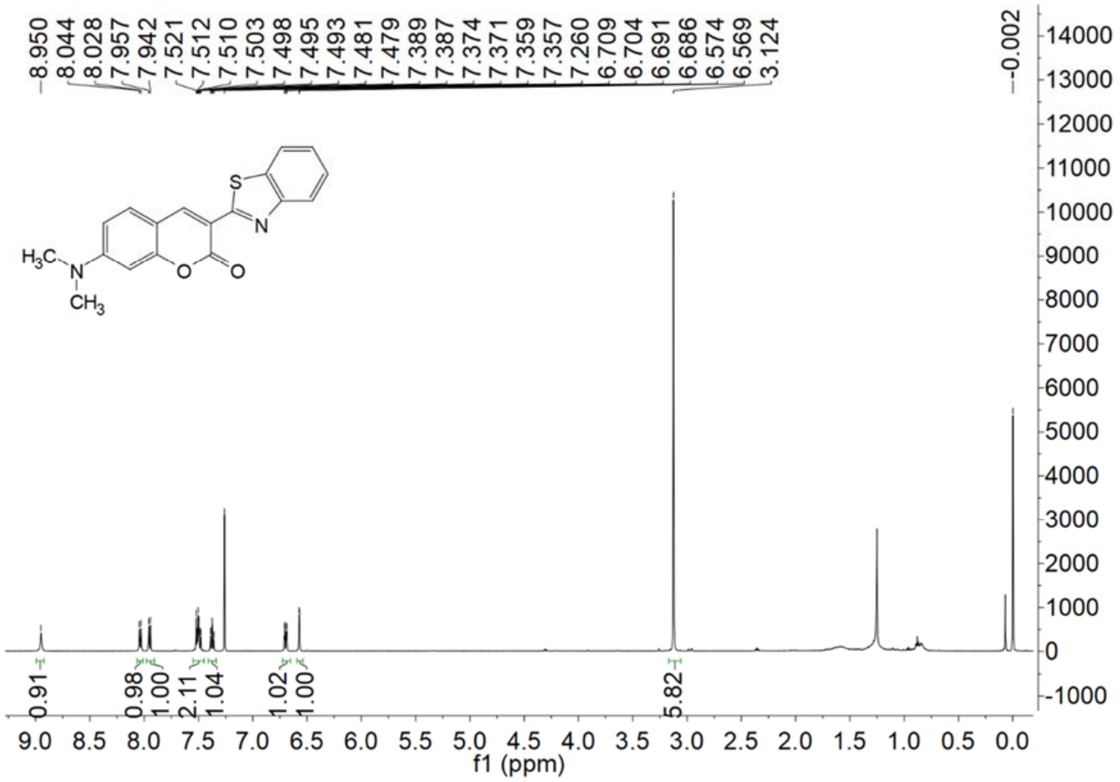

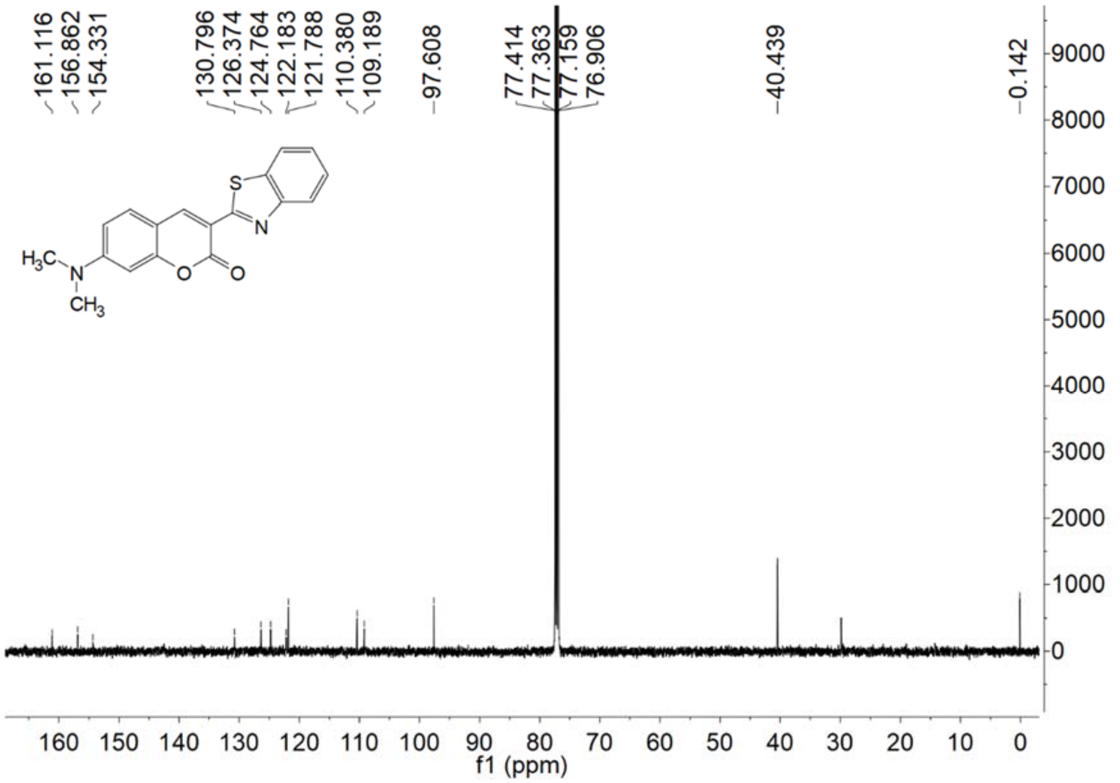

^{1}H and ^{13}C NMR of AH-3

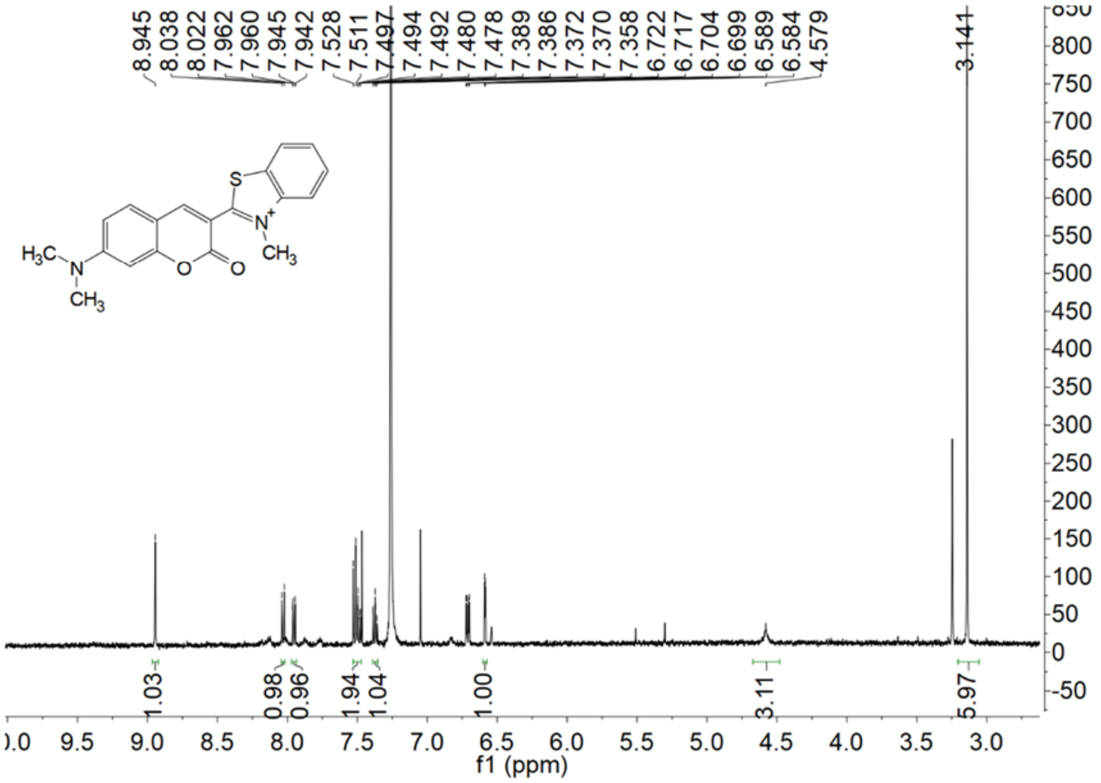

^{1}H NMR of AH-4

HRMS spectra traces of the probes

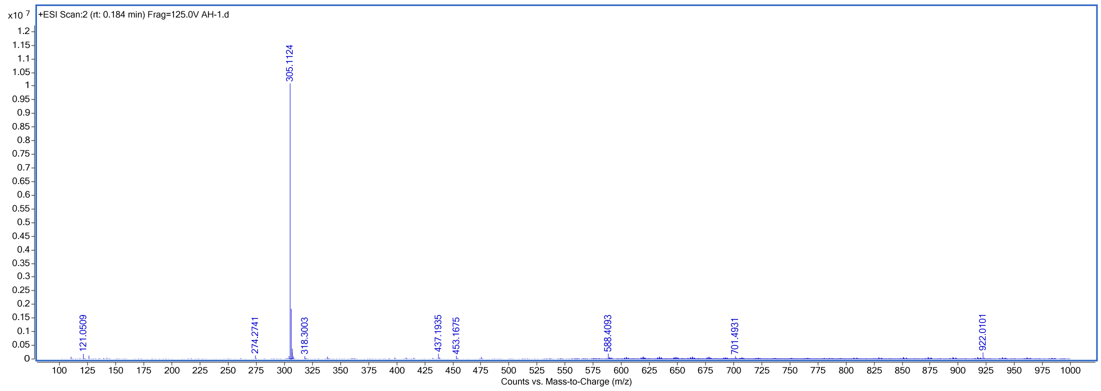

HRMS spectra trace of AH-1

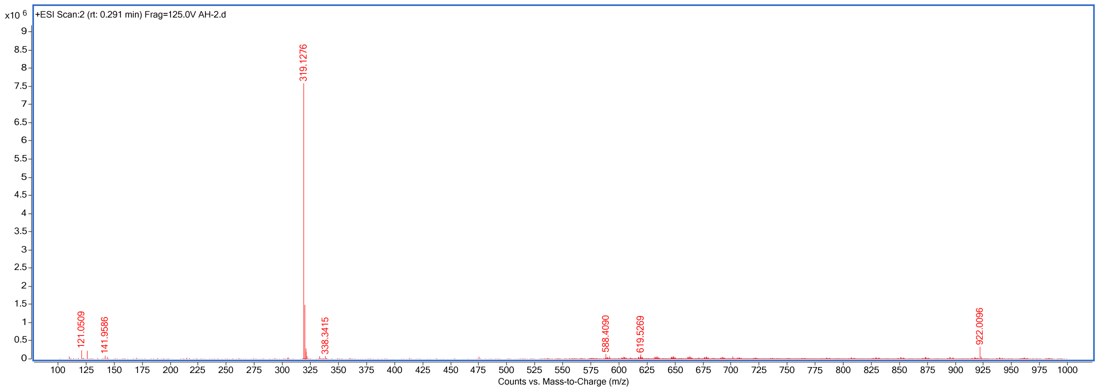

HRMS spectra trace of AH-2

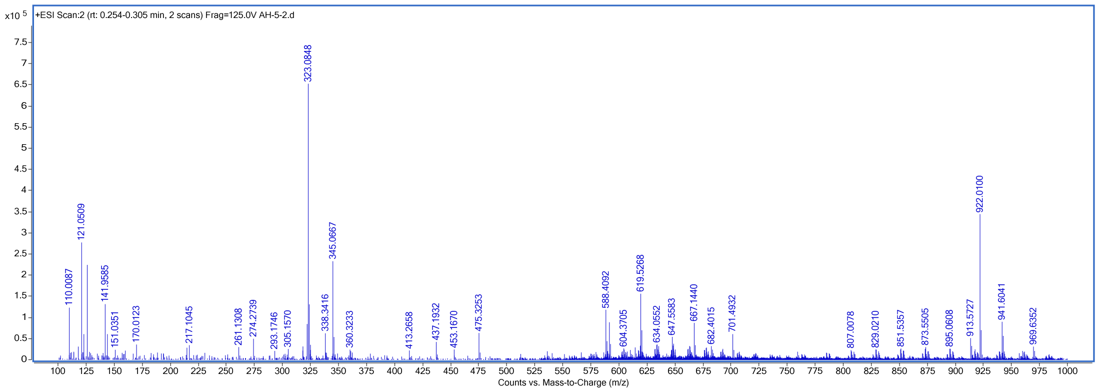

HRMS spectra trace of AH-3

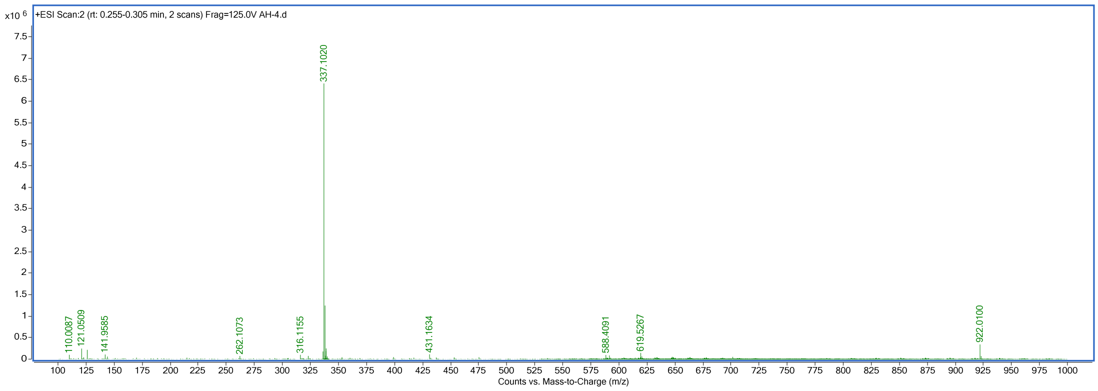

HRMS spectra trace of AH-4

References

1. Becke AD. DENSITY-FUNCTIONAL THERMOCHEMISTRY .3. THE ROLE OF EXACT EXCHANGE. Journal of Chemical Physics. 1993; 98: 5648-52.

2. M. J. Frisch GWT, H. B. Schlegel, G. E. Scuseria, M. A. Robb, J. R. Cheeseman, G. Scalmani, V. Barone, G. A. Petersson, H. Nakatsuji, X. Li, M. Caricato, A. V. Marenich, J. Bloino, B. G. Janesko, R.

Gomperts, B. Mennucci, H. P. Hratchian, J. V. Ortiz, A. F. Izmaylov, J. L. Sonnenberg, Williams, F. Ding, F. Lipparini, F. Egidi, J. Goings, B. Peng, A. Petrone, T. Henderson, D. Ranasinghe, V. G. Zakrzewski, J. Gao, N. Rega, G. Zheng, W. Liang, M. Hada, M. Ehara, K. Toyota, R. Fukuda, J. Hasegawa, M. Ishida, T. Nakajima, Y. Honda, O. Kitao, H. Nakai, T. Vreven, K. Throssell, J. A. Montgomery Jr., J. E. Peralta, F. Ogliaro, M. J. Bearpark, J. J. Heyd, E. N. Brothers, K. N. Kudin, V. N. Staroverov, T. A. Keith, R. Kobayashi, J. Normand, K. Raghavachari, A. P. Rendell, J. C. Burant, S. S. Iyengar, J. Tomasi, M. Cossi, J. M. Millam, M. Klene, C. Adamo, R. Cammi, J. W. Ochterski, R. L. Martin, K. Morokuma, O. Farkas, J. B. Foresman, D. J. Fox. Gaussian 16 Rev. C.01. Wallingford, CT, 2016. 2016.

3. Scalmani G, Frisch MJ, Mennucci B, Tomasi J, Cammi R, Barone V. Geometries and properties of excited states in the gas phase and in solution: theory and application of a time-dependent density functional theory polarizable continuum model. J Chem Phys. 2006; 124: 94107.
4. Chai JD, Head-Gordon M. Long-range corrected hybrid density functionals with damped atom-atom dispersion corrections. Phys Chem Chem Phys. 2008; 10: 6615-20.
5. Weigend F, Ahlrichs R. Balanced basis sets of split valence, triple zeta valence and quadruple zeta valence quality for H to Rn: Design and assessment of accuracy. Phys Chem Chem Phys. 2005; 7: 3297-305.
6. Marenich AV, Cramer CJ, Truhlar DG. Universal solvation model based on solute electron density and on a continuum model of the solvent defined by the bulk dielectric constant and atomic surface tensions. J Phys Chem B. 2009; 113: 6378-96.
7. Caricato M, Mennucci B, Tomasi J, Ingrosso F, Cammi R, Corni S, et al. Formation and relaxation of excited states in solution: a new time dependent polarizable continuum model based on time dependent density functional theory. J Chem Phys. 2006; 124: 124520.
8. Hanwell MD, Curtis DE, Lonie DC, Vandermeersch T, Zurek E, Hutchison GR. Avogadro: an advanced semantic chemical editor, visualization, and analysis platform. J Cheminform. 2012; 4: 17.
